# Differentiation of natural scrub communities of the *Cotoneastro-Amelanchieretum* group in Central Europe

**DOI:** 10.1371/journal.pone.0266868

**Published:** 2022-04-12

**Authors:** Krzysztof Świerkosz, Kamila Reczyńska

**Affiliations:** 1 Museum of Natural History, Faculty of Biological Sciences, University of Wrocław, Wrocław, Poland; 2 Department of Botany, Faculty of Biological Sciences, University of Wrocław, Poland; Instituto Federal de Educacao Ciencia e Tecnologia de Goias, BRAZIL

## Abstract

Most of Central European rocky scrub communities formed by *Cotoneaster integerrimus*, *Juniperus communis* and *Amelanchier ovalis* are included in the association *Cotoneastro-Amelanchieretum* (= *Junipero-Cotoneasteretum*). However, this leads to the creation of syntaxon whose internal diversity is so great that it seems necessary to examine validity of its existence in the current form. This diversity entails species composition, habitat requirements and geographical distribution. Therefore, we posed the following objectives: i) to investigate the variability of species composition of the rocky scrub; (ii) to determine if there are ecological differences between the communities distinguished by species variability; (iii) to determine the geographical ranges of individual syntaxa. Altogether we analyzed 387 phytosociological relevés from Central Europe. Vegetation types of rocky scrubs were identified using the unsupervised K-means algorithm and detrended correspondence analysis. Mean Ellenberg’s indicator values were applied to identify the environmental gradients shaping the plant communities. Obtained results confirmed the validity of dividing this broadly defined syntaxon into six distinct vegetation units. In order to present the studied communities in a broader context, we included into our analyses other rocky scrub with the occurrence of *Cotoneaster* sp. and *A*. *ovalis*, which formed the remaining three clusters. The observed differences in species composition were further supported by significant differences in soil reaction, temperature and continentality between the syntaxa. Moreover, the distinguished communities differed among one another in geographical range. Our study provides a new typology of the selected type of natural rocky scrub vegetation in Central Europe which involves environmental aspects, hence better reflects community-habitat relationships. This study also indicates a need for further revision of the classification of other types of natural scrub communities. Such classification should be based on modern data analysis methods and should primarily focus on lower vegetation units at pan-European scale.

## Introduction

The 21^st^ century has brought significant changes to research on diversity of plant communities worldwide. In the past, temporal and territorial constraints used to hinder the development of phytosociology. However, the last twenty years allowed vegetation scientists to examine vegetation beyond these constraints. One of the crucial improvements became vegetation databases which are commonly available and created at regional [1–4], pan-European [5], intercontinental [6, 7] and global scales [8]. Increasingly advanced numerical methods enable the analysis of large groups of relevés in a much more objective manner than in the early days of vegetation sciences. These modern tools enable the researcher to re-analyze community diversity not only at different levels of their organization, but also in any geographical area of interest. In recent years, these types of analyses have been conducted across Europe and referred to different plant communities. At regional level (i.e., within single countries or geobotanical units) examples include studies on diversity of beech forests [9–14], acido- and thermophilous oak forests [15–18], alluvial forests [19], fir and pine forests [20, 21], ravine forests [22] or wet and mesic meadows [23]. At the level of Central Europe, diversity of the following types of vegetation was re-examined: tall-herb communities [24], floodplain forests and alder carrs [25], oak-hornbeam forests [26], beech forests [27, 28], fen [29] and marsh [30] vegetation, mountain river gravel bars [31], lowland hay meadows [32] and others.

All the above-mentioned recent studies are continuously being integrated into the community classification system across Europe and are influencing the shape of the EUNIS (European Nature Information System) module of habitat classification. The aim of the latter is to provide a pan-European references of habitat units with a common unit description within a hierarchical classification. This approach is expected to fulfill objectives and support applications related to biodiversity monitoring and reporting at the European scale [33]. Noteworthy, all these studies contributed to the changes in the existing classification of plant communities at different spatial levels and extended our knowledge in this matter significantly. Moreover, research based on larger datasets from different regions allows us to see vegetation from a new and broader perspective.

In this context, syntaxonomic differentiation within the *Crataego-Prunetea* Tx. 1962 class in Europe was still not fully recognized at the time of this study. Although de Foucault and Julve [34] attempted to synthesize data on shrub communities from across Europe, their classification was not widely accepted. This was mainly due to the lack of clear definitions of communities, indication of groups of diagnostic species, and general descriptions of their ecology and geographical range. Therefore, while today we can be sure about the general features of European scrub communities, i.e., their ecological diversity [35] or the division of the *Crataego-Prunetea* class into orders and alliances [36], the diversity and distribution of the associations still leave many doubts, as evidenced by the latest syntaxonomic studies from Central and Western Europe [37–41].

The initial driving force behind the present study, was therefore, the need for the correct interpretation of the syntaxonomic affiliation of rocky scrub communities–characterized by the occurrence of *Cotoneaster integerrimus* and *Juniperus communis*–collected in southwestern Poland in the Sudetes Mountains (Sudetes Mts.). Since there are two names for this community in the literature–*Cotoneastro-Amelanchieretum* [42] and *Junipero-Cotoneasteretum* [43]–we wanted to establish which of them is currently valid.

However, during study of literature from Central Europe, we came to the realization that we faced a much more complex issue requiring more extensive analysis. It turned out that most of the relevés with the occurrence of *C*. *integerrimus* or *Amelanchier ovalis* were included in one out of two associations, regardless of their ecological or floristic differences. Meanwhile, the material analyzed to yield such classifications was collected at altitudes ranging from about 160 m to 1370 m a.s.l., and from vastly different rock substrates, in terms of fertility and chemistry. Consequently, relevés were marked by different ecological groups of species (e.g., acidophytes, xerophytes, calciphytes, subalpine species). Therefore, we considered that such deep internal differentiation would require detailed analysis, because the differences we observed between some of the described local phytocoenoses seemed to meet the criteria for separate associations.

We decided to analyze available phytosociological data (published and/or included in databases) from Central Europe, so far described as *Cotoneastero-Amelanchieretum* or *Junipero-Cotoneasteretum*. As a background for our analyses we used floristically and ecologically similar thermophilic rocky scrub communities i.e. *Cotoneastro integerrimi-Sorbetum chamaemespili*, *Erico carneae-Amelanchieretum ovalis* and *Waldsteinio geoidis-Spiraeetum mediae*, as well as the relevés of the *Pruno spinosae-Ligustretum vulgaris* subass. *cotoneasteretum integerrimi*.

In this study, we aim to determine if there are compositional and geographical differences between rocky scrub communities within the broad *Cotoneastro-Amelanchieretum* (= *Junipero-Cotoneasteretum*) group of phytocoenoses from Central Europe. Therefore, our main objectives are (i) to investigate the variability of species composition of the rocky scrub; (ii) to determine if there are ecological differences between the communities distinguished by species variability; (iii) to determine the geographical ranges of individual syntaxa. According to our knowledge, this is the first synthetic study of rocky scrub of Central Europe based on modern numerical methods.

## Materials and methods

### Phytosociological data and study area

Relevés with *A*. *ovalis*, *Cotoneaster* sp. dif. and *J*. *communis* were obtained from the Slovak Vegetation Database– 1338 relevés [2, 44]; Czech National Phytosociological database– 49 relevés [1]; Polish Vegetation Database– 126 relevés [3]; Austrian Vegetation Database– 18 relevés [4] and Hungarian Phytosociological Database COENODAT– 74 relevés [45] (see S1 Appendix for detail). We excluded two kinds of relevés from the initial dataset. These were relevés in which the cover of shrubs and trees was <25% and >25%, respectively. We did it because we assumed that the former represented rocky grassland communities, and the latter forest communities (including their initial form). We also removed relevés representing the subalpine communities of the *Roso pendulinae-Pinetea mugo* class as well as secondary shrubs with *J*. *communis* developing on the abandoned thermophilous grasslands.

We obtained additional 264 individual relevés of rocky shrub communities with the occurrence of *A*. *ovalis* and/or *Cotoneaster* sp. from literature sources [46–61] (see S2 Appendix). Additionally, we used our own material which was collected in Poland (25 relevés) according to Braun-Blanquet methods [62]. This material was preliminarily classified as *Junipero-Cotoneasteretum* [63] (see S3 Appendix). Polish relevés located in the protected areas (nature reserves) were collected with permission of the Regional Directorate for Environmental Protection in Wrocław (decision number WPN.6205.122.2017.MR). Some literature sources presented data exclusively or mainly in the form of synoptic tables [34, 50, 64, 65]. Due to the methodology adopted, we could not fully include them in the analyses.

**Fig 1:**
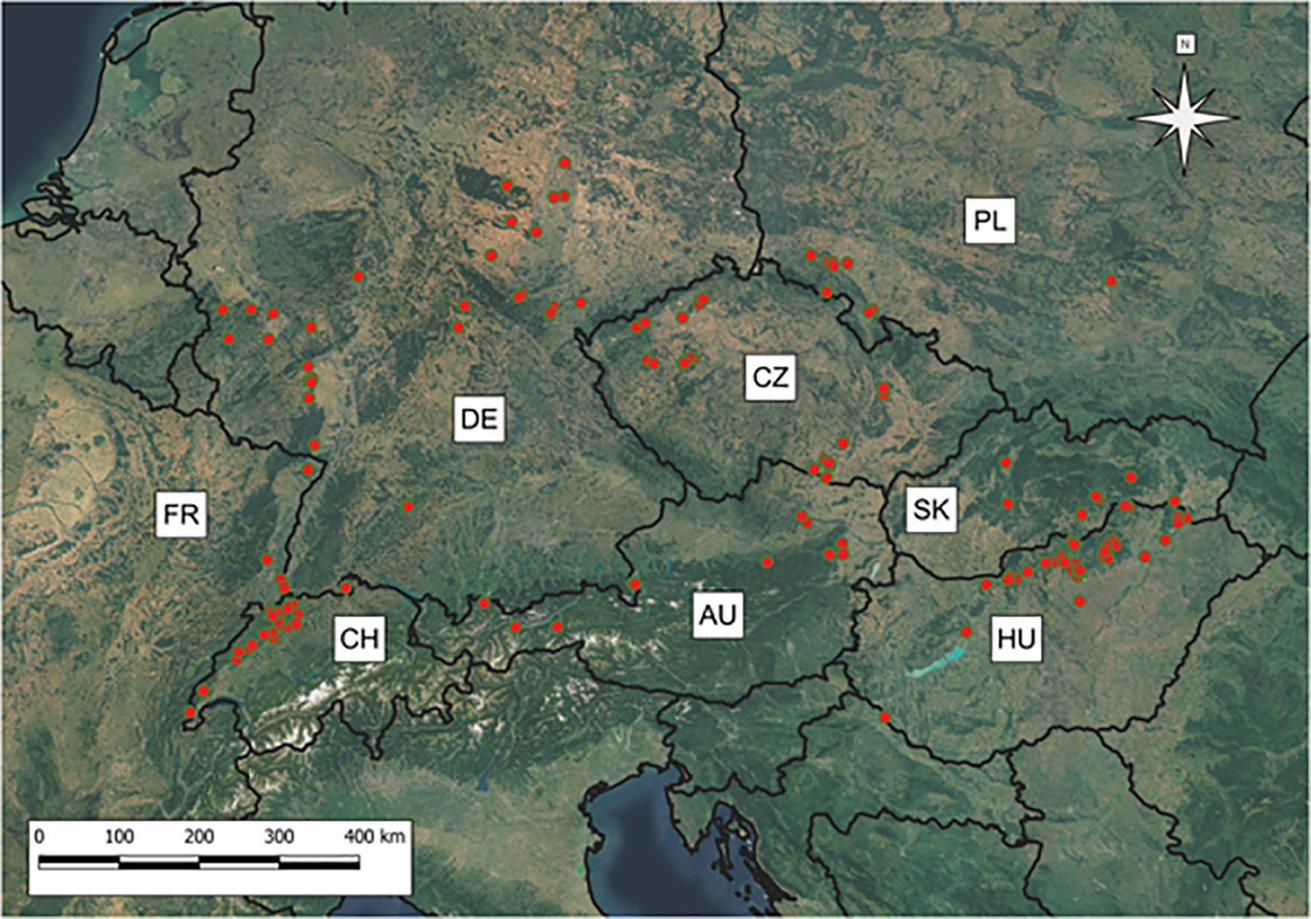
Location of study sites. Red dots indicate the distribution of the analyzed relevés of natural, rocky scrub communities across Central Europe (*n* = 387). Explanation of the abbreviations used: AU–Austria, CH–Switzerland, CZ–Czech Republic, DE–Germany, FR–France, HU–Hungary, PL–Poland, SK–Slovakia. Black contour lines–national boundaries. The map was made with Natural Earth. Free vector and raster map data @ naturalearthdata.com, ©MapTiler, ©OpenStreetMap contributors.

The final dataset of 387 relevés, which encompassed Central Europe (i.e., Slovakia, Czech Republic, Hungary, Poland, Germany, Austria, Switzerland and the eastern part of France), was the subject of final analyses (Fig 1).

### Data editing

Occurrences of the same woody species in different vertical layers were merged using the procedure implemented in the freeware JUICE 7.1 software–under the assumption that the overlap of layers was random [66, 67]. Moreover, some species were merged into aggregates (marked as ‘agg.’) based on Chytrý and Tichý [68] (see S4 Appendix for detail). Nomenclature follows Euro+ Med PlantBase [69] for vascular plants, and Ochyra et al. [70] for bryophytes. The nomenclature of higher vegetation units follows Mucina et al. [36].

### Phytosociological analyses

The vegetation types were identified using a K-means method with a power transformation implemented in JUICE 7.1 [66, 71]. The number of groups was determined *a posteriori* based on a scree plot of the values obtained in the crispness of classification analysis [72]. The latter was performed for different numbers of clusters expected (from 2 to 20) during the subsequent analyses. Finally, the division of relevés into nine, ecologically coherent groups, was optimal.

To characterize the distinguished communities, a diagnostic role of species was determined using the Φ coefficient for clusters of equalized size as a measure of fidelity [10, 73, 74]. Species with Φ *≥* 30 (0.3×100), constancy *≥* 10%, constancy ratio [75] higher than 1.5 and significant concentration in a particular cluster, tested by the Fisher’s exact test (*p* < 0.05), were considered to be diagnostic. Species with Φ *≥* 30 but the constancy ratio lower than 1.5 were not considered diagnostic. They usually occurred in several clusters, which decreased their role as determinants for communities within the studied type of vegetation. Constant species were defined as species with frequency of at least 40% in a given group of relevés. Distribution maps of the recognized communities were prepared using the mapping software DMAP 7.5 [76].

### Ecological analyses

Since many of the analyzed relevés lacked basic environmental data (i.e., topographic factors and/or edaphic conditions), the ecological differences between clusters were tested using Ellenberg’s indictor values (EIVs) [77]. We used EIVs which were corrected by Berg et al. [78], with reference to values of continentality, and supplemented by Chytrý et al. [79] with data on species not included previously. Out of the total number of 697 taxa, 31 species (4.4%) with south-eastern geographical range (i.e., Pontic-Pannonian and sub-Mediterranean-Pontic species) remained unclassified. EIVs weighted by percentage species’ cover were calculated for each relevé using the JUICE 7.1 software [66].

Due to the lack of consistent data on variation in habitat factors, it was not possible to use any of the constrained ordination analyses. Only part of the phytosociological relevés included in the present study has data on altitude (325 relevés, but part of these data was extrapolated based on the nearest known topographic point), slope (296 relevés) or aspect (304 relevés). Therefore, the variability of habitat conditions was visualized indirectly in CANOCO 5.0 [80] using detrended correspondence analysis (DCA) [81] (gradient length 5.76). The EIVs were passively projected into the DCA diagram with an awareness of the limitations of this method [82]. One of the weaknesses is that at the supra-regional scale, some of the indices are averaged to the center of the species’ range. The effect is that populations occurring on the edge of this range may show shifts on the scale of factors specified in the EIVs [83]. Additionally, the direct use of the EIVs in numerical analyses leads to collinearity, which precludes the use of some numerical methods such as canonical correspondence analysis (CCA), DCA and one-way analysis of variance (ANOVA) [82]. Also, the use of point estimates as a basis for inference does not consider variance among species EIVs within sampled plots and gives equal weighting to means calculated from plots with differing numbers of species [84]. However, regardless of the above-mentioned limitations, this method is still highly useful in the analysis of the diversity of vegetation at a supra-regional scale [85]. These limitations can be removed by applying mean randomized EIVs and modified permutation test [82].

Modified permutation test with 499 unrestricted permutations was conducted to identify the statistical significance of correlations (using Spearman’s coefficient) between the DCA sample scores obtained from CANOCO 5.0 and mean EIVs for relevés. The test was performed with *MoPeT_v1*.*2*.*r* script [82] in R software [86]. Permutational analysis of variance (one-way ANOVA on the mean randomized EIVs) and modified permutation test (with 499 unrestricted permutations) were also calculated using *MoPeT_v1*.*2*.r [82], to determine which of the EIVs differentiated the selected communities. Using ANOVA based on randomized values is an alternative to other tests under non-normal conditions, because it does not operate under the assumption of normality and uses actual scores [87].

## Results

### Phytosociological analysis

**Fig 2:**
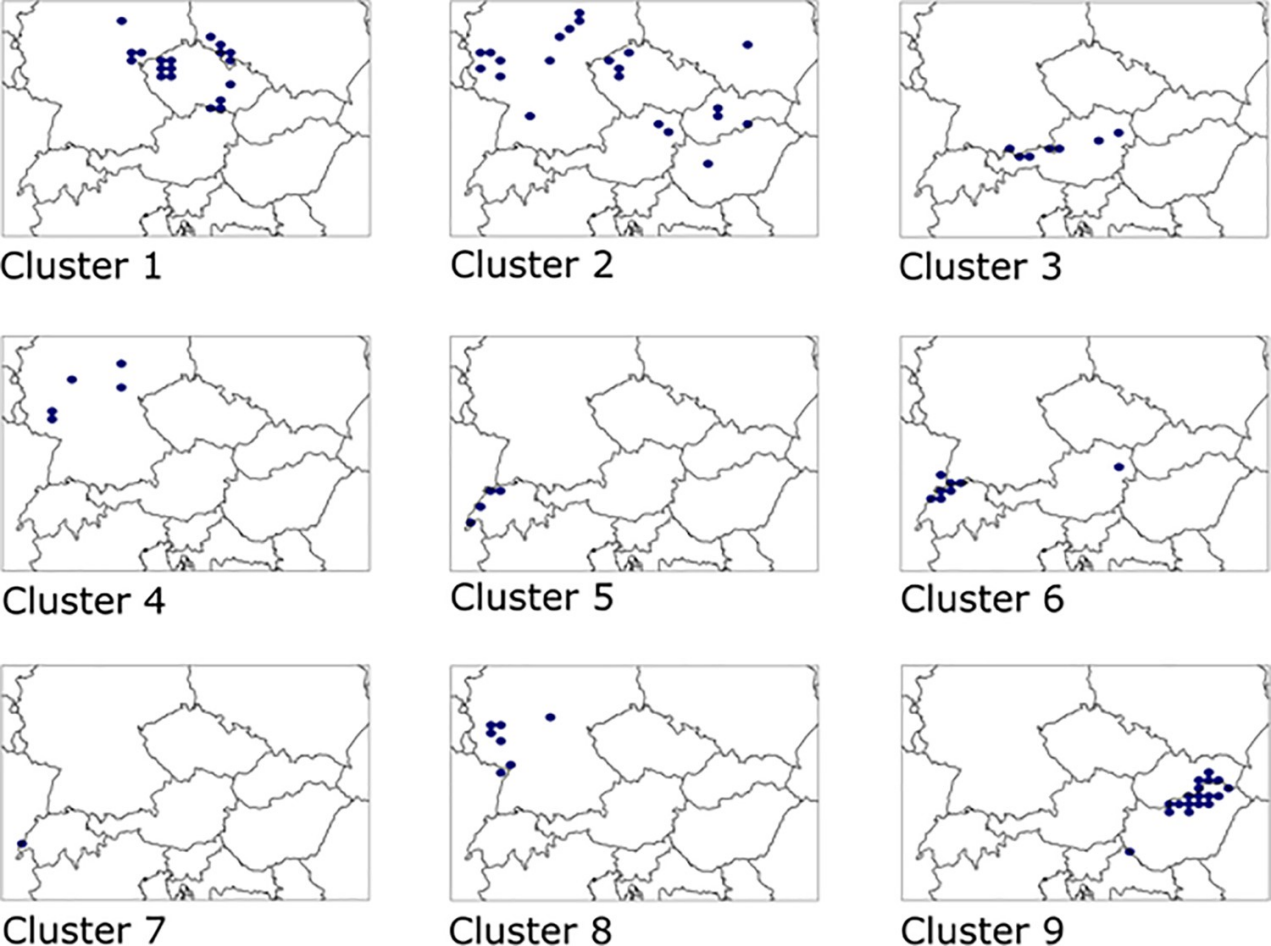
Distribution of the natural rocky scrub communities across Central Europe. Cluster 1 –*Cytiso scoparii-Cotoneasteretum integerrimi*; Cluster 2 –*Cotoneastro integerrimi-Amelanchieretum ovalis*; Cluster 3 –*Erico carneae-Amelanchieretum ovalis*; Cluster 4 –*Calluno vulgaris-Amelanchieretum ovalis*; Cluster 5 –comm. *Cotoneaster tomentosus-Amelanchier ovalis* sensu Moor 1979; Cluster 6 –*Coronillo emeri-Prunetum mahaleb*; Cluster 7 –*Cotoneastro integerrimi-Sorbetum chamaemespili*; Cluster 8 –*Pruno spinosae-Ligustretum vulgaris* subass. with *Cotoneaster integerrimus*; Cluster 9 –*Waldsteinio geoidis-Spiraeetum mediae*. Maps were prepared using mapping software DMAP 7.5 [76].

The studied rocky scrub communities with the presence of *Cotoneaster* sp., *Juniperus communis* and/or *Amelanchier ovalis* showed a great habitat and geographical diversity. We distinguished nine types of communities (Fig 2, S5 Appendix), six of which contained relevés originally described by various authors as the *Cotoneastro-Amelanchieretum* or *Junipero-Cotoneasteretum* associations, but only five may be treated as belonging to the *Cotoneastro-Amelanchieretum* s. lato. The nine associations are briefly characterized below. Their detailed descriptions, including synonyms of the names, specification of diagnostic and constant species, and additional notes on their nomenclature, are provided in S6 Appendix.

### Shrub communities from *Cotoneastro-Amelanchieretum* s. lato

*Cluster 1*. *Cytiso scoparii-Cotoneasteretum integerrimi Stöcker 1962*. This association has been reported so far only from the Bohemian Massif (Czech Republic, Poland, Germany) (Fig 2). It usually occurs at altitudes between 100 m and 670 m a.s.l. Within the distribution range it develops over bedrocks of almost lime-free, yet moderately nutrient-rich (basalt, greenschist, trachyte, andesite, diabase, some kinds of shales). This xerothermic shrub community is characterized by the loose structure and the presence of open rocky outcrops. Noteworthy among the diagnostic species is the occurrence of rocky grassland species common to the *Alysso-Festucion pallentis* alliance (e.g., *Asplenium septentrionale*, *Aurinia saxatilis*, *Festuca pallens*, *Hieracium schmidtii*), succulents (*Hylotelephium maximum*, *Jovibarba globifera* subsp. *globifera*) and species preferring moderately acidophilous and dry habitats (*Lembotropis nigricans*, *Vincetoxicum hirundinaria Calamagrostis arundinacea*). In the shrub layer *Cotoneaster integerrimus* is the most abundant, and regularly accompanied by *Rosa canina* agg. Importantly, *Amelanchier ovalis* is completely missing here. On the other hand, common occurrence of *Cytisus scoparius* in *locus classicus*, mentioned in the original name of the association, is a local phenomenon rather than a general rule. In the analyzed set of data this species has no diagnostic value (S5 Appendix, col. 1). However, most of the species which occurred in the Table 10 in Stöcker [48] are common in other sites from which this association is reported. Significantly, all the Stöcker’s [48] relevés belong to this cluster. Moreover, the ecological character of this species assemblage seems to be distinct and coherent.

*Cluster 2*. *Cotoneastro integerrimi-Amelanchieretum ovalis Faber ex Th*. *Müller 1966*. Phytocoenoses included in this cluster occur throughout Central Europe (Germany, Czech Republic, Austria, Poland, Hungary, Slovakia) (Fig 2), at altitudes between 100 m and 575 m (max. 700 m) a.s.l. They prefer calcareous habitats which derived from dolomites, limestones and marbles. In the group of diagnostic species there are mainly calcicolous and thermophilous taxa of the class *Festuco-Brometea* Br.-Bl. et Tx. ex Soó 1947 (*Anemone sylvestris*, *Asperula cynanchica*, *Centaurea scabiosa*, *Cirsium acaulon*, *Erysimum crepidifolium*, *Salvia pratensis*, *Scabiosa columbaria*). The shrub layer mainly consists of *Cotoneaster integerrimus* and *Rosa canina* agg., with an admixture of *Amelanchier ovalis*. The latter does not have natural sites in Poland and the Czech Republic. Hence, phytocoenoses with similar species composition and occurring in these two countries, have used to be included in the former *Junipero-Cotoneasteretum* association.

*Cluster 3*. *Erico carneae-Amelanchieretum ovalis Passarge 1997*. This cluster represents phytocoenoses from the Eastern Alps (Austria, Germany) (Fig 2), occurring in the middle mountain zone (between 950 m and 1100 m a.s.l.). They prefer consolidated and shallow rendzinas, which results in their high species richness. A distinctive feature of this association is the co-occurrence of species from different ecological groups. Therefore, in the herb layer there are calcicolous species of mountain grasslands (*Buphthalmum salicifolium*, *Calamagrostis varia*, *Carex alba*, *Erica carnea*, *Galium anisophyllon*, *Globularia cordifolia*, *Phyteuma orbiculare*, *Polygala chamaebuxus*, *Teucrium montanum*), forest species of the class *Carpino-Fagetea* Jakucs ex Passarge 1968 (*Cephalanthera damasonium*, *Hepatica nobilis*, *Melica nutans*, *Prenanthes purpurea*, *Salvia glutinosa*, *Valeriana tripteris*) as well as species typical of xerothermic grasslands of the *Festuco-Brometea* class (e.g, *Bromus erectus*, *Carex flacca*, *Festuca ovina* agg., *Hippocrepis comosa*, *Sesleria caerulea* agg., *Trifolium montanum*). The shrub layer is diversified and consists of *Amelanchier ovalis*, *Berberis vulgaris*, *Corylus avellana*, *Picea abies*, *Pinus mugo* and *Sorbus aria* agg. Importantly, *Cotoneaster integerrimus* rarely occurs here.

*Cluster 4*. *Calluno vulgaris-Amelanchieretum ovalis Rauschert (1969) 1990*. The association is known only from the central part of the Germany (Fig 2), where it prefers habitats derived from acidophilous and poor in nutrients bedrocks (Devonian and Ordovician clay shales, porphyry). It occurs at altitudes between 190 m and 450 m a.s.l., which indicates its upland character. The herb layer is dominated by vascular plants and mosses typical of acidophilous grasslands of the *Hyperico perforati-Scleranthion perennis* Moravec 1967 alliance (*Agrostis vinealis*, *Avenella flexuosa*, *Ceratodon purpureus*, *Genista pilosa*, *Hieracium umbellatum*, *Polytrichum piliferum*, *Rumex acetosella*). The mentioned species are accompanied by other taxa representing rocky grasslands (e.g., *Festuca heteropachys*, *F*. *lemanii*, *Teucrium scorodonia*). The shrub layer is formed by *Amelanchier ovalis*, *Rosa canina* agg., *Frangula alnus*, *Quercus petraea* agg. and *Betula pendula*. Noteworthy, this association is marked by the lowest soil reaction and continentality, against the background of all distinguished communities. Soil reaction as used here means the acidity or alkalinity of soil (soil pH). However, we did not measure soil pH in the field and we only relied on proxy data in the form of EIVs. Therefore, we use the term soil reaction as used by the source, Ellenberg [77]

*Cluster 5*. *comm*. *Cotoneaster tomentosus-Amelanchier ovalis sensu Moor 1979*. This community has been reported so far only from the Jura Mts. (France, Switzerland) (Fig 2), where it occurs at altitudes between 450 m and 860 m a.s.l. It prefers calcareous soils and south-facing slopes. Among the diagnostic species, highly abundant are shrubs and low trees which are of montane and sub-Mediterranean character. Besides *Amelanchier ovalis* this community is formed by *Cotoneaster tomentosus*, *Lonicera alpigena*, *Laburnum anagyroides*, *Rhamnus alpina*, *Taxus baccata*, *Sorbus mougeotii* and *Viburnum lantana*. Other shrub and low tree species are also abundant (*Berberis vulgaris*, *Juniperus communis* agg., *Lonicera xylosteum*, *Rhamnus cathartica*, *Rosa pendulina*, *Sorbus aria* agg., *S*. *aucuparia*, *Pinus sylvestris*), which makes it one of the most diverse communities within all described in this study. The herb layer is marked by the co-occurrence of calcicolous species (e.g., *Carduus defloratus* agg., *Sesleria caerulea* agg.) and species of montane meadows (*Laserpitium latifolium*, *Leucanthemum vulgare* agg., *Gentiana lutea*, *Galium mollugo* agg.). According to the original description by Moor [51], this community was included in the *Cotoneastro-Amelanchieretum* s. lato, as two distinct subassociations. For this reason, and due to the scarce phytosociological material, we did not decide to describe new association and left this issue for further analysis based on wider material.

#### Relevés originally assigned to the ass. *Cotoneastro-Amelanchieretum*, but not belonging to this association

*Cluster 6*. *Coronillo emeri-Prunetum mahaleb Gallandat 1972*. Relevés belonging to this association, originally classified as *Cotoneastro-Amelanchieretum*, come from Switzerland and France (45 relevés in Moor [51]) as well as Austria (4 relevés from NSG Glaslauterriegel-Heferlberg) (Fig 2). It is a calcicolous community occurring at altitudes between 400 m and 820 m a.s.l. on south-facing slopes (S, SW). Although *Amelanchier ovalis* and *Cotoneaster tomentosus* are present here, species diagnostic for the *Coronillo emeri-Prunetum mahaleb* dominate–it especially refers to *Hippocrepis emerus* and *Prunus mahaleb*. The composition of the shrub layer indicates a moderately thermophilous character of this association. Among shrub and low tree species we can observe here *Berberis vulgaris*, *Cornus sanguinea*, *Corylus avellana*, *Crataegus laevigata* agg., *C*. *monogyna* agg., *Fraxinus excelsior*, *Ligustrum vulgare*, *Lonicera xylosteum*, *Malus sylvestris* agg., *Rhamnus alpina*, *R*. *cathartica*, *Prunus spinosa*, *Quercus petraea* agg., *Rosa glauca*, *Sorbus aria* agg. and *Viburnum lantana*. In the herb layer, the occurrence of species typical of thermophilous forest fringe and tall-herb vegetation, such as *Polygonatum odoratum*, *Teucrium chamaedrys*, *Vincetoxicum hirundinaria* or *Viola hirta* is noticeable.

#### Associations with the regular occurrence of *Cotoneaster integerrimus* and/or *Amelanchier ovalis*, outside the range of *Cotoneastro-Amelanchieretum* s. lato

*Cluster 7*. *Cotoneastro integerrimi-Sorbetum chamaemespili Gillet in Gallandat et al*. *1995*. This association is known from the Alps, in Switzerland and on the German/Austrian border (Fig 2). It occurs on calcicolous lapiaz, sometimes also in spruce stands on lapiaz and in rocky pastures. It is therefore a primarily heliophilous and relatively thermophilic association, developing mainly on the south-facing slopes between 900 m and 1350 m a.s.l. The shrub layer consists of *Salix appendiculata*, *Sorbus chamaemespilus*, *S*. *aria*, *Picea abies*, *Rosa pendulina*, *Lonicera alpigena*, *L*. *caerulea* and *L*. *nigra* which indicate its high montane character. A rare component of the shrub layer is *Amelanchier ovalis*, whereas *Cotoneaster integerrimus* and *Juniperus communis* agg. are very common. In comparison to other communities presented, this association prefers the coolest temperature conditions.

*Cluster 8*. *Pruno spinosae-Ligustretum vulgaris Tüxen 1952 subass*. *with Cotoneaster integerrimus Korneck 1974*. Phytosociological relevés which form this cluster are marked by the high abundance of *C*. *integerrimus* and *A*. *ovalis* and were collected in Germany and France (Fig 2). However, the actual range of this association, within which it achieves its full variability, covers the whole of Central Europe. It is particularly common in lowlands and colline landscapes where it develops on mesic up to dry habitats. Although in most sites phytocoenoses of this cluster form secondary scrub, in places with rocky outcrops, they may be treated as a natural type of vegetation. In the latter case, the *Pruno-Ligustretum* is marked by the presence of moderately thermophilous and mesophilous shrub and low tree species such as *Euonymus europaeus*, *Ligustrum vulgare*, *Prunus avium*, *P*. *spinosa*, *Pyrus communis* agg., and *Ribes alpinum* which are accompanied by *Amelanchier ovalis*, *Cotoneaster integerrimus*, *Crataegus monogyna* agg., *Rhamnus cathartica*, *Rosa canina* agg. or *Quercus petraea*.

*Cluster 9*. *Waldsteinio geoidis-Spiraeetum mediae Zólyomi 1936*. The distribution range of this association is limited to the western part of Central Europe (Hungary, Slovakia, eastern Austria) (Fig 2). It prefers dry and skeletal soils such as rendzinas or rankers. It usually occurs at altitudes from 300 m to 750 m a.s.l. In Hungary, for example, it has been reported also from forest edges or even ruderal habitats, where nutrient-demanding species occur in its composition. The shrub layer is formed by *Spiraea media* with a constant presence of *Cotoneaster melanocarpus* and *Euonymus verrucosus*. Diagnostic species in the herb layer are mainly of Pontic-Pannonian and sub-Mediterranean character (*Allium flavum*, *Glechoma hirsuta*, *Iris variegata*, *Festuca pseudodalmatica*, *Lactuca viminea*, *Jovibarba globifera* ssp. *hirta*, *Poa pannonica*, *Rostraria cristata* or, *Waldsteinia geoides*). Therefore, this association is marked by the highest continentality index.

### Ecological differentiation

**Fig 3:**
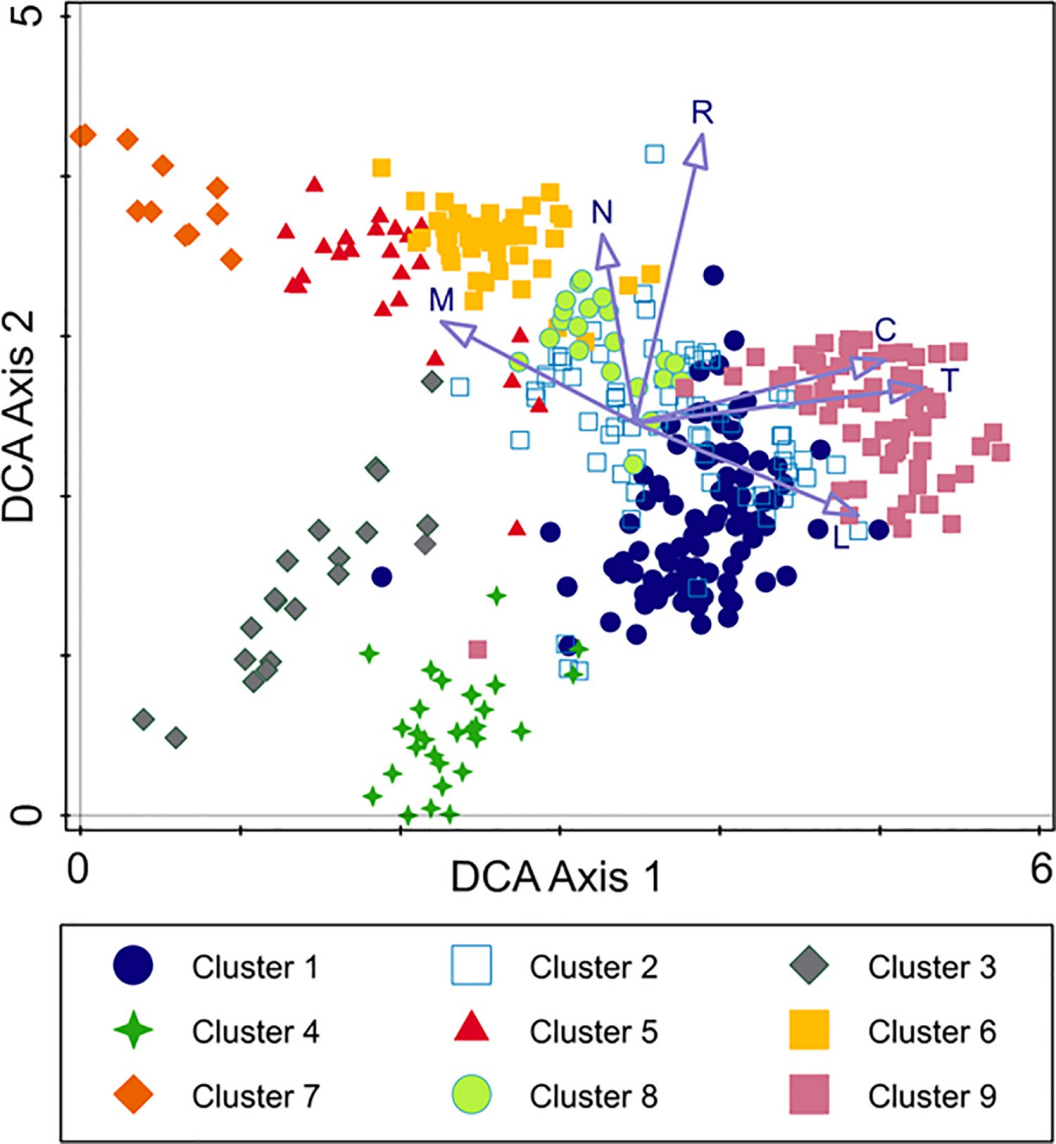
DCA diagram for the 387 vegetation plots of Central European rocky scrub communities with the passive projection of the mean Ellenberg’s indicator values (EIVs). Explanation: L–EIV for light; T–EIV for temperature; C–EIV for continentality; R–EIV for soil reaction; M–EIV for moisture; N–EIV for nutrients. Clusters 1–5 represent relevés once included in the *Cotoneastro-Amelanchieretum* s.l. but due to differences in species composition, habitat conditions and geographical range should be treated as separate associations. Cluster 6 represents relevés originally assigned to the ass. *Cotoneastro-Amelanchieretum*, but not belonging to this unit. Clusters 7–9 represent phytocoenoses with a regular occurrence of *Cotoneaster integerrimus* and/or *Amelanchier ovalis* outside the range of the *Cotoneastro-Amelanchieretum* s.l.. The numbers of clusters are compatible with Fig 2.

The DCA ordination diagram (Fig 3) shows a species compositional pattern within all distinguished rocky scrub associations. Total variation is 24.1%, supplementary variables account for 7.47% (adjusted explained variation is 6.01%). The DCA results revealed that the first and the second DCA axes explained 2.55% and 1.84% of compositional variability of studied communities, respectively. The first DCA axis was significantly correlated with the EIVs for continentality and temperature (both *p* < 0.01) as well as light and moisture (both *p* < 0.05). This indicates that the distinguished communities are distributed in a wide range of ecological factors. In the left part of the DCA diagram, plots which developed under higher moisture conditions and lower light availability (clusters 5–7) are concentrated. In turn, in the right part of the diagram xerothermic, well-lit and more continental communities are distributed (cluster 9). The second DCA axis was significantly correlated with the EIVs for soil reaction (*p* < 0.01) and nutrients (*p* < 0.05) (Table 1). This indicates that there are differences between the communities resulting from the type of bedrock and soil. Communities are distributed along the second axis from acidophilous ones (cluster 4) through those developing on effusive rocks and some types of sedimentary rocks (cluster 1, partly cluster 3), to calcareous communities with high soil reaction and better availability of nutrients (the remaining clusters).

**Table 1:** Significance of Spearman’s rank correlation of mean Ellenberg’s indicator values (EIVs) with two main DCA axes within rocky scrub communities of Central Europe. The statistical significance of correlation between studied plots and the EIVs is expressed by the *p*–values based on modified permutation test (P.modif).

	AXIS 1 [AX1]		AXIS 2 [AX2]	
	rho estimate	P.modif	rho estimate	P.modif
Ellenberg _Temperature_	0.775	0.004	-0.012	0.940
Ellenberg _Continentality_	0.842	0.004	-0.046	0.868
Ellenberg _Moisture_	-0.523	0.012	0.171	0.440
Ellenberg _Light_	0.530	0.020	-0.308	0.156
Ellenberg _Soil Reaction_	-0.048	0.904	0.701	0.004
Ellenberg _Nutrients_	-0.160	0.448	0.504	0.016

**Fig 4:**
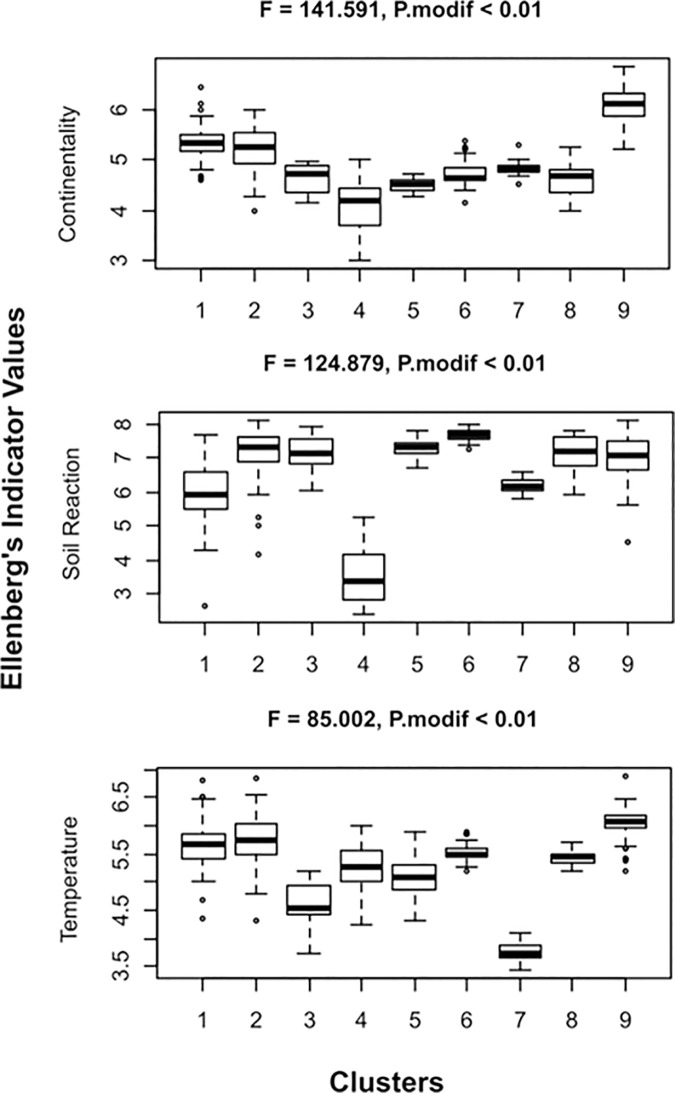
Summary box-and-whisker plots of mean Ellenberg’s indicator values (EIVs) for clusters recognized within rocky scrub communities of Central Europe produced by one-way ANOVA based on the mean randomized EIVs. The central line of each box indicates the median value, box boundaries the lower (25%) and upper (75%) quartiles, and whiskers the range of values. P. modif. was calculated using a modified permutation test of significance for analysis of mean randomized EIVs; F–variance ratio statistic. Numbers of clusters are the same as in Fig 2.

ANOVA on the mean randomized EIVs for the nine relevé groups suggested that soil reaction, continentality and temperature (all *p* < 0.01) played the most significant role in shaping the diversity of the studied vegetation types (Fig 4).

## Discussion

According to the analyses of phytosociological relevés from all over Central Europe, rock scrubs with *Cotoneaster* sp. diff., *Amelanchier ovalis* and *Juniperus communis* are more diverse than previously thought. These communities grow on different types of rocks, from highly acidophilic to calcicolous, and at different elevations. Thus, their differentiation presented in this paper concerns both the species composition and the preferred edaphic conditions.

The first mention about rocky scrub communities involving *C*. *integerrimus* appeared in Firbas and Sigmond [88]. They described fragments of the *Cotoneaster integerrima-Polygonatum odoratum* community which had developed on the boulder sites on the top of Donnersberge in Böhmischen Mittelgebirge (now Milešovka, 836.5 m a.s.l.). The community consisted of *Festuca pallens*, *Rosa canina*, *R*. *glauca*, *Iris aphylla*, *Stachys recta*, *Poa nemoralis* and *Digitalis ambigua* (= *D*. *grandiflora*). Unfortunately, its characterization was devoid of phytosociological documentation. Therefore, it could not be the basis for a description of the association. Still, for historical reasons, it should be noted and emphasized.

The rocky shrub community with *C*. *integerrimus* and *A*. *ovalis* was documented for the first time by Faber [46], who presented 7 relevés of the community *Cotoneaster-Amelanchier*-Gesträuch from White (Weisse) Jura. This community was transitional in character between typical scrub communities and rocky grasslands. Shrub species in only four relevés had significant abundance–the remaining three relevés represented rocky outcrops with high proportions of chasmophytes and low abundance of shrubs and they were excluded from our analyses (see Table 1 in [46]). In the following years, the communities of typical rocky shrubs were described under the name proposed by Faber, to which the study of Tüexen [89] made a particular contribution, and consequently the name of the association was often quoted as *Cotoneastero integerrimi-Amelanchieretum* Tüxen 1952, *Cotoneastro integerrimi-Amelanchieretum ovalis* Faber (1936) Tx. 1952 or *Cotoneastro-Amelanchieretum* Faber 1936 ex Tx. 1952 [42, 43].

However, these names were not given in accordance with the code of nomenclature [90]. Therefore, *Cotoneastro-Amelanchieretum* Faber ex Th. Müller 1966 was considered to be the correct name in the course of an extensive review of the naming of the associations distinguished in Germany–precisely owing to proper diagnosis made by Müller (see footnote 658 in [42]). Since then, communities with both shrub species and recorded throughout Central Europe, from the foothills to the subalpine zone and from different types of bedrocks [50–56], have been described under this name. Moreover, in almost all national synthetic studies this name was considered correct [64, 65, 91–93]. Only Passarge [58] did not use it with regard to mountain phytocoenoses from Upper Bavaria (Germany), with the occurrence of *Erica carnea*, *Pinus mugo* or *Polygala chamaebuxus* describing the new association *Erico carneae-Amelanchieretum*.

The second name, *Junipero-Cotoneasteretum*, which is used in literature for the studied scrubs, was proposed by Hoffmann [47]. He published 8 relevés (see Table 4 in Hoffmann [47]) referring to the community, somewhat similar to the one described earlier by Faber [46] in terms of ecological character. However, Hoffmann’s community was marked by the co-dominance of *Juniperus communis* and *Sorbus aria* in the place of *A*. *ovalis* and constituting typical xerothermic shrubs on steep, south-facing slopes. This name was adopted as the correct one in the Czech nomenclature [43], probably due to the lack of natural sites of *A*. *ovalis* in the Czech Republic. In this country also the *Polygonato-Sorbetum ariae* prov [54] association was provisionally described–presently it is treated as the synonym of the *Junipero-Cotoneasteretum* [43]. Apart from the Czech Republic, the latter name is nowadays either considered the synonym of the *Cotoneastro-Amelanchieretum* [42] or not mentioned at all in synthetic studies [65, 93].

The researcher who noticed greater variability within *Cotoneastro-Amelanchieretum* s. lato was Rauschert [57]. On the basis of differences in species composition he divided this large unit into two main groups. Among the communities with the dominance of *A*. *ovalis* ("Die Felsbirnengebüsche") and *C*. *integerrimus* („Zwergmispelgebüsche”) he distinguished three and five associations, respectively. In the first group there were: *Calluno-Amelanchieretum* Rauschert (69) ass. nov., *Erysimo-Amelanchieretum* Rauschert (69) ass. nova and *Cotoneastro-Amelanchieretum* Faber 36. In the second group Rauschert included: *Seslerio-Cotoneasteretum* Rauschert (69) ass. nov, *Junipero-Cotoneasteretum* Hofmann 58, *Roso ellipticae-Cotoneasteretum* Rauschert (69) ass. nov, *Sarothamno scoparii- Cotoneasteretum* Stöcker 62 and *Lembotropido-Cotoneasteretum* (Niemann 62) Rauschert ass. nov.). Currently, all the above-mentioned names are treated as synonyms either of the *Cotoneastro-Amelanchieretum* in most of the Central European countries e.g. [42] or *Junipero-Cotoneasteretum* in the Czech Republic [43].

The furthest reaching deliberations in this matter were presented by de Foucault and Julve [34] who distinguished in Europe a separate alliance *Amelanchierion ovalis* Arlot 1985 (currently the name is synonymous with the *Berberidion vulgaris* Br.-Bl. ex Tx. 1952, (comp. Mucina et al. [36]) and further divided it into three suballiances. They listed a total of 14 associations: 11 of which had in their name the part „*Amelanchieretum ovalis”* (including one “community with *Amelanchier ovalis*”), and the name of remaining three had the segment „*Cotoneasteretum integerrimae”*. However, the lack of descriptions of the syntaxa, maps of distribution and designation of holotypic relevés do not allow to refer to the results presented in de Foucault and Julve [34] in a reliable way.

Our research confirms the need for revision of the existing approaches to the syntaxonomic units within studied rocky shrub communities. It turns out that the current classification of this type of vegetation is too general, especially at the level of lower vegetation units (such as associations), and does not fully correspond with their ecological and geographic diversity. The internal differentiation that we initially observed in the phytosociological material was confirmed by the results of the research, both in terms of floristic composition and ecological differentiation. Each of the distinguished units has a separate group of diagnostic species and differs from the others in terms of habitat characteristics–bedrock type, soil reaction and continentalism index. The latter indicates such climate features as annual temperature amplitudes or annual precipitation variability. Within the communities described so far as *Cotoneastro-Amelanchieretum* and *Junipero-Cotoneasteretum*, six groups of relevés were distinguished. The remaining three groups formed the background against which the analyzed material was examined. The number of relevés originally assigned by their authors to different syntaxonomical units against the clusters distinguished in this study is presented in Table 2.

**Table 2:** The number of relevés originally assigned by their authors to different syntaxonomical units in the clusters distinguished in this study. The numbers of clusters are compatible with Fig 2.

Cluster number	1	2	3	4	5	6	7	8	9
No of relevés	95	68	20	28	24	49	11	21	71
*Cytiso scoparii-Cotoneasteretum integerrimi* Stöcker 1962	12	.	.	.	.	.	.	.	.
*Lembotropido-Cotoneasteretum* Rauschert 1990	8	.	.	.	.	.	.	.	.
*Polygonato-Sorbetum ariae* Kolbek et Petříček 1985 prov	7	.	.	.	.	.	.	.	.
*Junipero communis-Cotoneasteretum integerrimi* Hofmann 1958	68	20	.	.	.	.	.	.	.
*Cotoneastro integerrimi-Amelanchieretum ovalis* Faber ex Th. Müller 1966	.	11	5	22	24	46	.	.	.
*Roso elipticae-Cotoneasteretum* Rauschert 1990	.	10	.	.	.	.	.	.	.
*Cotoneaster-Amelanchier*-Gesträuch Faber 1938	.	4	.	.	.	.	.	.	.
*Erysimo-Amelanchieretum* Rauschert 1990	.	5	.	.	.	.	.	.	.
*Seslerio-Cotoneastretum* Rauschert 1990	.	11	.	.	.	.	.	.	.
*Erico-Amelanchieretum ovalis* Passarge 1997	.	.	12	.	.	.	.	.	.
*Festuco rupicolae-Juniperetum sabinae* Exner 2004	.	.	1	.	.	.	.	.	.
*Calluno-Amelanchieretum* Rauschert 1990	.	.	.	6	.	.	.	.	.
*Cotoneastro integerrimi-Sorbetum chamaemespili* Gillet in Gallandat et al. 1995	.	.	.	.	.	.	11	.	.
*Pruno spinosae-Ligustretum vulgaris* Tüxen 1952 subass. with *Cotoneaster integerrimus*	.	.	2	.	.	.	.	21	.
*Waldsteinio geoidis-Spiraeetum mediae* Zólyomi 1936	.	.	.	.	.	.	.	.	70
*Prunetum fruticosae* Dziubałtowski 1925	.	.	.	.	.	1	.	.	
Unassigned in databases	.	7		.	.	2	.	.	1

Cluster 1 consists of relevés which have been initially assigned to four different associations, with the majority of phytocoenoses described as *Junipero-Cotoneasteretum* in Germany, the Czech Republic and Poland (including material collected by us). We decided to assign all relevés from this group to the *Cytiso scoparii-Cotoneasteretum integerrimi* Stöcker 1962 association, because it is the earliest of the correctly used names; while the original relevés of Hofmann [47] are included in the second group, along with other phytocoenoses of limestone bedrocks. It should be emphasized that in Polish phytosociological literature the occurrence of this association was ignored [94], until 2015 [63].

For the relevés in the second cluster we kept the original name *Cotoneastro integerrimi-Amelanchieretum ovalis* indicated by Rennwald et al. [42]. In this group, relevés initially assigned to six different associations (mainly *Junipero-Cotoneasteretum*, *Cotoneastro-Amelanchieretum* and *Seslerio-Amelanchieretum*) were included (Table 2). Moreover, both the original community described by Faber [46] and relevés of the typical *Junipero-Cotoneasteretum* [47] are the components of this group, which indicates that differences between these two communities are not sufficient for distinguishing them in the rank of separate associations.

Cluster 3 justifies the distinction of a separate *Erico carneae-Amelanchieretum* association, because it contains all the relevés published by Passarge [58] and additional five relevés from Tauern and Eastern Alps, originally included in the *Cotoneastro-Amelanchieretum* [53, 56]. Although the original diagnosis of Passarge places this phytocoenoses at altitude of more than 950 m a.s.l., other relevés shift the lower range limit of the association to 520 m a.s.l.

Cluster 4 represents the phytocoenoses described by Rauschert [57] as *Calluno vulgaris-Amelanchieretum ovalis*. Analysis of the collected material showed that relevés of this community were also under the name *Cotoneastro integerrimi-Amelanchieretum* published by Korneck [50] from porphyry and Devonian shales near Lahntal (Middle Hesse) and from some localities in Rhenish Hesse.

Relevés included in the cluster 5 were published by Moor [51] and their species composition do not resemble any of the phytocoenoses described so far. However, we decided that it would be premature to describe the new association on the basis of relevés from one mountain range and we kept this group in the rank of the community *Cotoneaster tomentosus-Amelanchier ovalis* sensu Moor 1979. Although Passarge [58] classified these phytocoenoses as the *Cotoneastro tomentosi-Amelanchieretum* Moor 1979, he did not indicate a type relevé. Moreover, the mentioned name was earlier used by Jakucs [95] and referred to Mediterranean and relict-like, highland scrub. This association was reported from the Vértes Mts. and Keszthely Mts. in the Central Transdanubian region in Hungary. It consisted of *Amelanchier ovalis*, *Cotoneaster tomentosa*, *Cotinus coggygria*, *Carpinus orientalis*, *Fraxinus ornus*, as well as species of rocky grasslands and steppe grassy slopes, developing on soils derived from dolomite bedrock [96]. Also the name proposed by de Foucault and Julve [34]: *Coluteo arborescentis-Amelanchieretum ovalis* (Moor 1979) ass. nov. hoc loco refers to the phytocoenoses of this group. However, in Table 17–1, col. 8, by de Foucault and Julve [34], where the diversity of communities with *A*. *ovalis* is presented, it is difficult to find a clear distinctiveness between proposed associations.

The set of diagnostic species of the cluster 6 indicates its affiliation to the *Coronillo emeri-Prunetum mahaleb* association with a high proportion of *A*. *ovalis*. In the preliminary analyses carried out for the purposes of this study, we also included the original relevés of this association from the study of Gallandat [49] in the comparisons, and they combined with other relevés into one homogenous group. However, the first description of the *Coronillo*-*Prunetum* ([49], Table 1, rel. 12–43) contains information only on the species composition of the shrub layer, so we could not use this data in the final version of the article, let alone to identification of species diagnostic for each group.

Relevés which gathered in the remaining three clusters of scrubs (7–9) did not pass to other groups previously classified as the *Cotoneastro*-*Amelanchieretum* s. lato. Thus, there are grounds to believe that all the syntaxonomic units we have distinguished within the studied group of scrub have the character of independent associations or communities, because they have been divided at the same level as well-developed and accurately described syntaxa.

## Conclusions

Until now most of Central European rocky scrub communities formed by *Cotoneaster* sp., *J*. *communis* and *A*. *ovalis* were classified more frequently as the *Cotoneastro-Amelanchieretum* or less often as the *Junipero-Cotoneasteretum* associations. Consequently, this led to the creation of syntaxon in which the diversity of phytocoenoses in terms of species composition, ecological requirements or even the geographical range cast doubt on their continued existence in this broad sense.

Thus, our study systematizes the knowledge about the diversity of rocky scrub communities originally included in this large *Cotoneastro-Amelanchieretum* group of phytocoenoses. The obtained results have confirmed the validity of dividing this broadly defined syntaxon into distinct vegetation units–five in the rank of the association and one in the rank of the community. In the present approach, the typology of studied communities is based not only on species composition, but also the differences in habitat preferences and geographical distribution. Hence, the associations distinguished in this way became more coherent and, due to involving environmental aspects, better reflecting community-habitat relationships. Therefore, the scrub community classification presented here is also more useful in understanding the diversity of natural habitat types in Europe, in the context of their biodiversity and protection requirements. On the other hand, our study should be treated as an initial step towards further revision of the classification of natural scrub communities in Europe which ought to be based on modern data analysis tools. Moreover, future classification should be conducted with special reference to lower vegetation units such as associations, and should obligatory correspond with ecological requirements of distinguished syntaxa.

## Supporting information

S1 Appendix ID numbers of relevés obtained from databases and analyzed in this study. (PDF)

S2 Appendix Relevés obtained from literature sources. (XLSX)

S3 Appendix Authors’ relevés from the Sudetes Mts. (TXT)

S4 Appendix List of aggregates used in the analyses. (PDF)

S5 Appendix Summarized synoptic table with percentage frequency and fidelity values of Central European rocky scrub communities (387 relevés). (PDF)

S6 Appendix Detailed descriptions of distinguished syntaxa. (PDF)
