# Supplementary material for: Differentiation of natural scrub communities of the Cotoneastro-Amelanchieretum group in Central Europe
Source: PLoS One. 2022 Apr 12;17(4):e0266868. doi: 10.1371/journal.pone.0266868 (PMC9004749; doi:10.1371/journal.pone.0266868)
Supplement: S1 Appendix — (PDF) [file pone.0266868.s001.pdf]

Differentiation of natural scrub communities of *Cotoneastro-Amelanchieretum* group in Central Europe

Świerkosz K., Reczyńska K.

Appendix S1
ID numbers of relevés obtained from external databases and used in the analyses

Database	Relevé number
Austrian Vegetation Database	35000
Austrian Vegetation Database	35003
Austrian Vegetation Database	35011
Austrian Vegetation Database	35012
Austrian Vegetation Database	35350
Austrian Vegetation Database	35607
Austrian Vegetation Database	35619
Austrian Vegetation Database	36648
Austrian Vegetation Database	307509
Austrian Vegetation Database	307510
Austrian Vegetation Database	402301
Austrian Vegetation Database	402302
Austrian Vegetation Database	402303
Austrian Vegetation Database	402336
Czech National Phytosociological Database	102018
Czech National Phytosociological Database	102199
Czech National Phytosociological Database	102200
Czech National Phytosociological Database	102201
Czech National Phytosociological Database	102202
Czech National Phytosociological Database	102203
Czech National Phytosociological Database	102204
Czech National Phytosociological Database	102205
Czech National Phytosociological Database	102206
Czech National Phytosociological Database	102207
Czech National Phytosociological Database	102208
Czech National Phytosociological Database	102209
Czech National Phytosociological Database	102210
Czech National Phytosociological Database	102211
Czech National Phytosociological Database	102212
Czech National Phytosociological Database	102213
Czech National Phytosociological Database	102214
Czech National Phytosociological Database	102215
Czech National Phytosociological Database	102216
Czech National Phytosociological Database	102217
Czech National Phytosociological Database	102218
Czech National Phytosociological Database	111842
Czech National Phytosociological Database	111843
Czech National Phytosociological Database	111844
Czech National Phytosociological Database	203568
Czech National Phytosociological Database	203569
Czech National Phytosociological Database	347153

Czech National Phytosociological Database	347154
Czech National Phytosociological Database	347323
Czech National Phytosociological Database	347326
Czech National Phytosociological Database	347425
Czech National Phytosociological Database	400350
Czech National Phytosociological Database	400746
Czech National Phytosociological Database	401320
Czech National Phytosociological Database	401321
Czech National Phytosociological Database	401322
Czech National Phytosociological Database	412511
Czech National Phytosociological Database	412512
Czech National Phytosociological Database	412513
Czech National Phytosociological Database	412514
Czech National Phytosociological Database	455079
Czech National Phytosociological Database	455087
Czech National Phytosociological Database	455088
Czech National Phytosociological Database	455089
Czech National Phytosociological Database	546261
Hungarian Phytosociological Database	204380
Hungarian Phytosociological Database	204381
Hungarian Phytosociological Database	207061
Hungarian Phytosociological Database	207062
Hungarian Phytosociological Database	207068
Hungarian Phytosociological Database	207069
Hungarian Phytosociological Database	207070
Hungarian Phytosociological Database	207071
Hungarian Phytosociological Database	207072
Hungarian Phytosociological Database	207073
Hungarian Phytosociological Database	207074
Hungarian Phytosociological Database	207084
Hungarian Phytosociological Database	207085
Hungarian Phytosociological Database	207086
Hungarian Phytosociological Database	207089
Hungarian Phytosociological Database	207090
Hungarian Phytosociological Database	207095
Hungarian Phytosociological Database	207096
Hungarian Phytosociological Database	207097
Hungarian Phytosociological Database	207098
Hungarian Phytosociological Database	207099
Hungarian Phytosociological Database	207100
Hungarian Phytosociological Database	207101
Hungarian Phytosociological Database	207102
Hungarian Phytosociological Database	207103
Hungarian Phytosociological Database	207104
Hungarian Phytosociological Database	207105
Hungarian Phytosociological Database	207106
Hungarian Phytosociological Database	207107
Hungarian Phytosociological Database	207108
Hungarian Phytosociological Database	207308
Hungarian Phytosociological Database	207309

Hungarian Phytosociological Database	207310
Hungarian Phytosociological Database	207311
Hungarian Phytosociological Database	207312
Hungarian Phytosociological Database	207313
Hungarian Phytosociological Database	207314
Hungarian Phytosociological Database	207315
Hungarian Phytosociological Database	207316
Hungarian Phytosociological Database	207317
Hungarian Phytosociological Database	207318
Hungarian Phytosociological Database	207319
Hungarian Phytosociological Database	207320
Hungarian Phytosociological Database	207321
Hungarian Phytosociological Database	207322
Hungarian Phytosociological Database	207323
Hungarian Phytosociological Database	207324
Hungarian Phytosociological Database	207325
Hungarian Phytosociological Database	207326
Hungarian Phytosociological Database	211017
Hungarian Phytosociological Database	211018
Hungarian Phytosociological Database	211019
Hungarian Phytosociological Database	211020
Polish Vegetation Database	17
Polish Vegetation Database	151
Polish Vegetation Database	913
Polish Vegetation Database	916
Polish Vegetation Database	4271
Polish Vegetation Database	4272
Polish Vegetation Database	4273
Polish Vegetation Database	4274
Polish Vegetation Database	4275
Polish Vegetation Database	11766
Polish Vegetation Database	29724
Polish Vegetation Database	29725
Slovak Vegetation Database	25
Slovak Vegetation Database	97
Slovak Vegetation Database	99
Slovak Vegetation Database	186
Slovak Vegetation Database	195
Slovak Vegetation Database	231
Slovak Vegetation Database	631
Slovak Vegetation Database	207087
Slovak Vegetation Database	207088
Slovak Vegetation Database	207091
Slovak Vegetation Database	207092
Slovak Vegetation Database	207093
Slovak Vegetation Database	207094
