# Supplementary material for: Differentiation of natural scrub communities of the Cotoneastro-Amelanchieretum group in Central Europe
Source: PLoS One. 2022 Apr 12;17(4):e0266868. doi: 10.1371/journal.pone.0266868 (PMC9004749; doi:10.1371/journal.pone.0266868)
Supplement: S4 Appendix — (PDF) [file pone.0266868.s004.pdf]

Differentiation of natural scrub communities of *Cotoneastro-Amelanchieretum* group in Central Europe

Świerkosz K., Reczyńska K.

Appendix S4

The full list of aggregates used in the analyses. Aggregates determined by databases from which the relevés were obtained are marked in bold.

Achillea millefolium agg. (A. collina, A. distans, A. pannonica)

Campanula rotundifolia agg. (C. xylocarpa, C. gentilis)

Centaurea stoebe s. lat. (incl. C. micranthos)

Crduus defloratum (incl. C. crassifolius)

Dactylis glomerata agg. (incl. D. polygama)

Dorycnium pentaphyllum agg. (incl. D. germanicum)

Festuca ovina agg. (incl. F. firmula)

Festuca stricta (incl. subsp. sulcata)

G. pumilum agg.

G. pusillum agg.

Galeobdolon luteum agg.

Galium aparine agg.

Galium intermedium agg. (G. schultesi, G, sylvaticum)

Galium mollugo agg. (incl. G. album)

Genista tinctoria (incl. subsp. elatior)

Geranium robertianum agg. (incl. G. purpureum)

Helianthemum nummularium agg. (incl. H nummu.larium subsp. obscurum, H. grandiflorum, Helianthemum ovatum)

Hypnum cupressiforme agg. (incl. H. lacunosum)

Knautia arvensis agg.

Koeleria pyramidata agg.

Leucanthemum vulgare agg. (incl. Leucanthemum adustum, L. maximum)

Lotus corniculatus agg. (incl. L. borbasi, L. tenuis)

Luzula campestris agg. (incl. L. pallescens, L. multiflora, L. sudetica)

Malus sylvestris agg.

Melica ciliata agg. (incl. M. transsilvanica)

Mellitis melissophyllum agg. (incl. M. carpatica)

Plagiomnium affine agg. (P. elatum, P. ellipticum, P. medium)

Poa pratensis agg. (incl. Poa angustifolia)

Primula veris agg.

Pseudolysimachion spicatum agg. (inlc. P. orchideum, P. spicatum)

Pyrus communis agg. (incl. P. pyraster)

Quercus petraea agg. (incl. Q. dalechampii)

Quercus pubescens agg.

Ranunculus polyanthemos agg. (incl. R. polyanthemos subsp. nemorosus)

Rosa canina agg. (incl. R. dumalis, Rosa vosagiacea)

Rosa tomentosa agg.

Senecio nemorensis agg. (incl. Senecio ×decipiens Senecio ×futakii, Senecio germanicus Senecio hercynicus, Senecio ovatus)

Sesleria caerulea agg

Solidago virgaurea agg.

Sorbus aria agg. (incl. S. danubialis)

Thalictrum minus agg.

Thesium linophyllon agg. (incl. T. bavarum)

Thymus praecox agg.

Thymus pulegioides agg. (incl. T. pannonicus)

Torilis japonica agg.

Valeriana officinalis agg. (incl. ssp. collina)

Valeriana stolonifera agg. (incl. Valeriana wallrothii, V angusifolia)

Veronica chamaedrys agg. (incl. V vindobonensis)

Veronica hederifolia agg. (incl. V. sublobata)
