# Supplementary material for: Differentiation of natural scrub communities of the Cotoneastro-Amelanchieretum group in Central Europe
Source: PLoS One. 2022 Apr 12;17(4):e0266868. doi: 10.1371/journal.pone.0266868 (PMC9004749; doi:10.1371/journal.pone.0266868)
Supplement: S5 Appendix — (PDF) [file pone.0266868.s005.pdf]

Differentiation of natural scrub communities of *Cotoneastro-Amelanchieretum* group in Central Europe

Świerkosz K., Reczyńska K.

Appendix S5

Summarized synoptic table with percentage frequency and fidelity values of Central European rocky scrub communities (387 relevés). The Φ coefficient values (multiplied by 100) are presented as superscripts. Diagnostic species (Φ>30 and constancy ratio >1.5) are in bold. Among accompanying species the most common ones are included in the table. Letter "d" after species name indicates moss layer.

**Cluster No.**	**1**	**2**	**3**	**4**	**5**	**6**	**7**	**8**	**9**
**No. of relevés**	**95**	**68**	**20**	**28**	**24**	**49**	**11**	**21**	**71**
Cytiso scoparii-Cotoneasteretum integerrimi									
Festuca pallens	**60**$^{51.1}$	24^{---}	.$^{---}$	.$^{---}$	17^{---}	.$^{---}$	.$^{---}$	.$^{---}$	15^{---}
Hieracium schmidtii	**26**$^{49.3}$	.$^{---}$	.$^{---}$	.$^{---}$	.$^{---}$	.$^{---}$	.$^{---}$	.$^{---}$	.$^{---}$
Asplenium septentrionale	**37**$^{43.8}$	3^{---}	.$^{---}$	.$^{---}$	.$^{---}$	.$^{---}$	.$^{---}$	.$^{---}$	17^{---}
Aurinia saxatilis	**22**$^{41.6}$	.$^{---}$	.$^{---}$	.$^{---}$	.$^{---}$	.$^{---}$	.$^{---}$	.$^{---}$	3^{---}
Calamagrostis arundinacea	**27**$^{40.8}$	3^{---}	.$^{---}$	.$^{---}$	.$^{---}$	.$^{---}$	.$^{---}$	.$^{---}$	7^{---}
Lembotropis nigricans	**25**$^{37.9}$	4^{---}	.$^{---}$	.$^{---}$	4^{---}	2^{---}	.$^{---}$	.$^{---}$	.$^{---}$
Vincetoxicum hirundinaria	**74**$^{38.0}$	26^{---}	20^{---}	.$^{---}$	8^{---}	47^{---}	.$^{---}$	.$^{---}$	47^{---}
Jovibarba globifera globifera*	**12**$^{32.5}$	.$^{---}$	.$^{---}$	.$^{---}$	.$^{---}$	.$^{---}$	.$^{---}$	.$^{---}$	.$^{---}$
Cotoneastro integerrimi-Amelanchieretum ovalis									
Cirsium acaulon	2^{---}	**19**$^{39.0}$	.$^{---}$	.$^{---}$	.$^{---}$	.$^{---}$	.$^{---}$	.$^{---}$	.$^{---}$
Salvia pratensis	1^{---}	**25**$^{35.9}$	.$^{---}$	.$^{---}$	.$^{---}$	.$^{---}$	.$^{---}$	.$^{---}$	10^{---}
Thymus pulegioides agg.	22$^{18.6}$	**35**$^{36.0}$	.$^{---}$	.$^{---}$	.$^{---}$	.$^{---}$	.$^{---}$	.$^{---}$	11^{---}
Asperula cynanchica	7^{---}	**29**$^{35.7}$	.$^{---}$	.$^{---}$	.$^{---}$	.$^{---}$	.$^{---}$	.$^{---}$	14^{---}
Erysimum crepidifolium	$^{---}$	**16**$^{38.5}$	.$^{---}$	.$^{---}$	.$^{---}$	.$^{---}$	.$^{---}$	.$^{---}$	.$^{---}$
Centaurea scabiosa	.$^{---}$	**22**$^{34.9}$	5^{---}	.$^{---}$	.$^{---}$	2^{---}	.$^{---}$	.$^{---}$	3^{---}
Scabiosa columbaria	.$^{---}$	**15**$^{34.5}$	.$^{---}$	.$^{---}$	.$^{---}$	.$^{---}$	.$^{---}$	.$^{---}$	1^{---}
Anemone sylvestris	.$^{---}$	**12**$^{32.7}$	.$^{---}$	.$^{---}$	.$^{---}$	.$^{---}$	.$^{---}$	.$^{---}$	.$^{---}$
Erico-Amelanchieretum ovalis									
Erica carnea	.$^{---}$	.$^{---}$	**100**^{100}	.$^{---}$	.$^{---}$	.$^{---}$	.$^{---}$	.$^{---}$	.$^{---}$
Polygala chamaebuxus	1^{---}	1^{---}	**80**$^{86.7}$	.$^{---}$	.$^{---}$	.$^{---}$	.$^{---}$	.$^{---}$	.$^{---}$
Galium anisophyllon	.$^{---}$	.$^{---}$	**65**$^{79.1}$	.$^{---}$	.$^{---}$	.$^{---}$	.$^{---}$	.$^{---}$	.$^{---}$
Ranunculus polyanthemos	.$^{---}$	.$^{---}$	**65**$^{78.0}$	.$^{---}$	.$^{---}$	.$^{---}$	.$^{---}$	.$^{---}$	1^{---}
Potentilla erecta	.$^{---}$	.$^{---}$	**60**$^{75.8}$	.$^{---}$	.$^{---}$	.$^{---}$	.$^{---}$	.$^{---}$	.$^{---}$
Pinus mugo	.$^{---}$	.$^{---}$	**45**$^{65.1}$	.$^{---}$	.$^{---}$	.$^{---}$	.$^{---}$	.$^{---}$	.$^{---}$
Carex ornithopoda	.$^{---}$	.$^{---}$	**40**$^{61.2}$	.$^{---}$	.$^{---}$	.$^{---}$	.$^{---}$	.$^{---}$	.$^{---}$
Melica nutans	6^{---}	4^{---}	**55**$^{56.3}$	.$^{---}$	8^{---}	6^{---}	.$^{---}$	.$^{---}$	.$^{---}$

Calamagrostis varia	.$^{---}$	1^{---}	**40$^{56.1}$**	.$^{---}$	4^{---}	.$^{---}$	.$^{---}$	.$^{---}$	.$^{---}$
Phyteuma orbiculare	.$^{---}$	4^{---}	**35$^{52.8}$**	.$^{---}$	.$^{---}$	.$^{---}$	.$^{---}$	.$^{---}$	.$^{---}$
Cephalanthera damasonium	.$^{---}$	.$^{---}$	**30$^{52.8}$**	.$^{---}$	.$^{---}$	.$^{---}$	.$^{---}$	.$^{---}$	.$^{---}$
Hepatica nobilis	1^{---}	1^{---}	**30$^{50.0}$**	.$^{---}$	.$^{---}$	.$^{---}$	.$^{---}$	.$^{---}$	.$^{---}$
Trifolium montanum	.$^{---}$	1^{---}	**30$^{49.6}$**	.$^{---}$	.$^{---}$	.$^{---}$	.$^{---}$	.$^{---}$	1^{---}
Tortella tortuosa d	1^{---}	3^{---}	**30$^{48.5}$**	.$^{---}$	.$^{---}$	.$^{---}$	.$^{---}$	.$^{---}$	.$^{---}$
Buphthalmum salicifolium	.$^{---}$	1^{---}	**25$^{46.2}$**	.$^{---}$	.$^{---}$	.$^{---}$	.$^{---}$	.$^{---}$	.$^{---}$
Carex alba	.$^{---}$	.$^{---}$	**35$^{43.8}$**	.$^{---}$	12$^{9.9}$	4^{---}	.$^{---}$	.$^{---}$	.$^{---}$
Molinia caerulea agg.	.$^{---}$	.$^{---}$	**20$^{42.9}$**	.$^{---}$	.$^{---}$	.$^{---}$	.$^{---}$	.$^{---}$	.$^{---}$
Rhamnus saxatilis	.$^{---}$	4^{---}	**30$^{41.5}$**	.$^{---}$	.$^{---}$	8^{---}	.$^{---}$	.$^{---}$	.$^{---}$
Hippocrepis comosa	.$^{---}$	15$^{12.0}$	**35$^{41.4}$**	.$^{---}$	4^{---}	2^{---}	.$^{---}$	.$^{---}$	.$^{---}$
Carex flacca	.$^{---}$	4^{---}	**35$^{41.3}$**	.$^{---}$	17$^{14.7}$	.$^{---}$	.$^{---}$	.$^{---}$	.$^{---}$
Valeriana tripteris	.$^{---}$	1^{---}	**20$^{40.8}$**	.$^{---}$	.$^{---}$	.$^{---}$	.$^{---}$	.$^{---}$	.$^{---}$
Lotus corniculatus agg.	3^{---}	15$^{11.7}$	**35$^{40.8}$**	.$^{---}$	4^{---}	.$^{---}$	.$^{---}$	.$^{---}$	.$^{---}$
Prenanthes purpurea	.$^{---}$	.$^{---}$	**15$^{37.0}$**	.$^{---}$	.$^{---}$	.$^{---}$	.$^{---}$	.$^{---}$	.$^{---}$
Salvia glutinosa	.$^{---}$	.$^{---}$	**15$^{37.0}$**	.$^{---}$	.$^{---}$	.$^{---}$	.$^{---}$	.$^{---}$	.$^{---}$
Pseudoscleropodium purum d	.$^{---}$	.$^{---}$	**15$^{37.0}$**	.$^{---}$	.$^{---}$	.$^{---}$	.$^{---}$	.$^{---}$	.$^{---}$
Laserpitium siler	.$^{---}$	.$^{---}$	**20$^{35.7}$**	.$^{---}$	.$^{---}$	6$^{6.5}$	.$^{---}$	.$^{---}$	.$^{---}$
Teucrium montanum	.$^{---}$	9^{---}	**30$^{35.6}$**	.$^{---}$	4^{---}	.$^{---}$	.$^{---}$	.$^{---}$	10^{---}
Globularia cordifolia	.$^{---}$	4^{---}	**20$^{33.5}$**	.$^{---}$	4^{---}	.$^{---}$	.$^{---}$	.$^{---}$	.$^{---}$
Bromus erectus	.$^{---}$	13$^{13.7}$	**25$^{33.2}$**	.$^{---}$	.$^{---}$	.$^{---}$	.$^{---}$	.$^{---}$	4^{---}
Calluno-Amelanchieretum									
Avenella flexuosa	25$^{13.2}$	4^{---}	.$^{---}$	**82$^{73.6}$**	.$^{---}$	.$^{---}$	.$^{---}$	.$^{---}$	.$^{---}$
Calluna vulgaris	9^{---}	1^{---}	5^{---}	**57$^{62.8}$**	.$^{---}$	.$^{---}$	.$^{---}$	.$^{---}$	.$^{---}$
Teucrium scorodonia	.$^{---}$	3^{---}	.$^{---}$	**50$^{59.4}$**	.$^{---}$	.$^{---}$	.$^{---}$	10^{---}	.$^{---}$
Polytrichum piliferum d	2^{---}	.$^{---}$	.$^{---}$	**43$^{57.2}$**	.$^{---}$	.$^{---}$	.$^{---}$	5^{---}	.$^{---}$
Genista pilosa	.$^{---}$	9^{---}	10^{---}	**43$^{44.4}$**	.$^{---}$	.$^{---}$	.$^{---}$	10^{---}	1^{---}
Rubus fruticosus agg.	6^{---}	3^{---}	.$^{---}$	**39$^{42.3}$**	.$^{---}$	2^{---}	.$^{---}$	14^{---}	1^{---}
Festuca lemanii	.$^{---}$	.$^{---}$	.$^{---}$	**18$^{40.5}$**	.$^{---}$	.$^{---}$	.$^{---}$	.$^{---}$	.$^{---}$
Festuca heteropachys	.$^{---}$	1^{---}	.$^{---}$	**18$^{38.3}$**	.$^{---}$	.$^{---}$	.$^{---}$	.$^{---}$	.$^{---}$
Ceratodon purpureus d	16$^{16.6}$	1^{---}	.$^{---}$	**29$^{37.1}$**	.$^{---}$	.$^{---}$	.$^{---}$	.$^{---}$	.$^{---}$
Agrostis vinealis	.$^{---}$	.$^{---}$	.$^{---}$	**14$^{36.1}$**	.$^{---}$	.$^{---}$	.$^{---}$	.$^{---}$	.$^{---}$
Rumex acetosella	9$^{8.2}$	3^{---}	.$^{---}$	**25$^{34.5}$**	.$^{---}$	.$^{---}$	.$^{---}$	.$^{---}$	3^{---}
Hieracium umbellatum	5^{---}	3^{---}	.$^{---}$	**25$^{34.0}$**	.$^{---}$	2^{---}	.$^{---}$	5^{---}	1^{---}
Betula pendula	5^{---}	.$^{---}$	.$^{---}$	**18$^{33.9}$**	.$^{---}$	.$^{---}$	.$^{---}$	.$^{---}$	.$^{---}$
comm. Cotoneater tomentosus-Amelanchier ovalis									
Sorbus mougeotii	.$^{---}$	.$^{---}$	.$^{---}$	.$^{---}$	**71$^{66.0}$**	20$^{10.0}$	9^{---}	.$^{---}$	.$^{---}$
Carduus defloratus agg.	.$^{---}$	1^{---}	15^{---}	.$^{---}$	**58$^{55.4}$**	16$^{7.0}$	.$^{---}$	.$^{---}$	.$^{---}$
Leucanthemum vulgare agg.	15$^{9.1}$	10^{---}	.$^{---}$	.$^{---}$	**42$^{44.4}$**	2^{---}	.$^{---}$	.$^{---}$	.$^{---}$
Gentiana lutea	.$^{---}$	.$^{---}$	.$^{---}$	.$^{---}$	**21$^{43.8}$**	.$^{---}$	.$^{---}$	.$^{---}$	.$^{---}$
Arctostaphylos uva-ursi	.$^{---}$	.$^{---}$	.$^{---}$	.$^{---}$	**21$^{43.8}$**	.$^{---}$	.$^{---}$	.$^{---}$	.$^{---}$
Taxus baccata	.$^{---}$	.$^{---}$	.$^{---}$	.$^{---}$	**21$^{43.8}$**	.$^{---}$	.$^{---}$	.$^{---}$	.$^{---}$
Laserpitium latifolium	.$^{---}$	3^{---}	10^{---}	.$^{---}$	**38$^{39.6}$**	12$^{6.2}$	.$^{---}$	.$^{---}$	4^{---}
Laburnum anagyroides	.$^{---}$	.$^{---}$	.$^{---}$	.$^{---}$	**12$^{33.8}$**	.$^{---}$	.$^{---}$	.$^{---}$	.$^{---}$
Coronillo emeri-Prunetum mahaleb **var. with** ***Amelanchier ovalis***									
Hippocrepis emerus	.$^{---}$	1^{---}	.$^{---}$	.$^{---}$	33$^{18.5}$	**94$^{79.1}$**	.$^{---}$	.$^{---}$	.$^{---}$

Rosa glauca	$.^{---}$	$.^{---}$	$.^{---}$	$.^{---}$	12^{---}	**$61^{67.8}$**	$.^{---}$	$.^{---}$	$.^{---}$
Prunus mahaleb	1^{---}	$.^{---}$	$.^{---}$	11^{---}	4^{---}	**$63^{54.1}$**	$.^{---}$	19^{---}	11^{---}
Malus sylvestris agg.	3^{---}	4^{---}	$.^{---}$	$.^{---}$	$.^{---}$	**$43^{53.6}$**	$.^{---}$	5^{---}	$.^{---}$
Crataegus laevigata agg.	1^{---}	12^{---}	$.^{---}$	$.^{---}$	$.^{---}$	**$51^{49.5}$**	$.^{---}$	19^{---}	1^{---}
Cornus sanguinea	13^{---}	$31^{16.3}$	5^{---}	$.^{---}$	8^{---}	**$63^{48.3}$**	$.^{---}$	5^{---}	3^{---}
Fraxinus excelsior	12^{---}	19^{---}	30^{---}	$.^{---}$	4^{---}	**$65^{46.5}$**	$.^{---}$	10^{---}	3^{---}
Viola hirta	7^{---}	18^{---}	25^{---}	$.^{---}$	$33^{15.2}$	**$55^{35.3}$**	$.^{---}$	$.^{---}$	11^{---}
Cotoneastro integerrimi-Sorbetum chamaemespili									
Lonicera caerulea	$.^{---}$	$.^{---}$	$.^{---}$	$.^{---}$	$.^{---}$	$.^{---}$	**100^{100}**	$.^{---}$	$.^{---}$
Sorbus chamaemespilus	$.^{---}$	$.^{---}$	$.^{---}$	$.^{---}$	$.^{---}$	$.^{---}$	**$91^{94.9}$**	$.^{---}$	$.^{---}$
Lonicera nigra	$.^{---}$	$.^{---}$	$.^{---}$	$.^{---}$	$.^{---}$	$.^{---}$	**$73^{84.0}$**	$.^{---}$	$.^{---}$
Salix appendiculata	$.^{---}$	$.^{---}$	5^{---}	$.^{---}$	$17^{4.2}$	$.^{---}$	**$91^{82.9}$**	$.^{---}$	$.^{---}$
Picea abies	1^{---}	9^{---}	$45^{23.1}$	$.^{---}$	12^{---}	$.^{---}$	**$100^{72.2}$**	$.^{---}$	$.^{---}$
Rosa pendulina	$.^{---}$	3^{---}	10^{---}	$.^{---}$	$46^{27.6}$	$.^{---}$	**$82^{61.7}$**	$.^{---}$	4^{---}
Daphne mezereum	$.^{---}$	1^{---}	$.^{---}$	$.^{---}$	$17^{10.7}$	$.^{---}$	**$55^{59.6}$**	$.^{---}$	$.^{---}$
Sambucus racemosa	$.^{---}$	1^{---}	$.^{---}$	$.^{---}$	$.^{---}$	$.^{---}$	**$36^{56.8}$**	$.^{---}$	$.^{---}$
Salix caprea	1^{---}	$.^{---}$	$.^{---}$	$.^{---}$	$.^{---}$	$.^{---}$	**$27^{49.0}$**	$.^{---}$	$.^{---}$
Rubus idaeus	$12^{5.4}$	$.^{---}$	10^{---}	7^{---}	$.^{---}$	$.^{---}$	**$36^{38.3}$**	$.^{---}$	1^{---}
Pruno spinosae-Ligustretum vulgaris **subass. with** ***Cotoneaster integerrimus***									
Euonymus europaeus	11^{---}	3^{---}	$.^{---}$	$.^{---}$	$.^{---}$	10^{---}	$.^{---}$	**$48^{51.1}$**	$.^{---}$
Prunus spinosa	16^{---}	34^{---}	$.^{---}$	$.^{---}$	$.^{---}$	$55^{25.0}$	$.^{---}$	**$86^{49.7}$**	24^{---}
Prunus avium	2^{---}	$.^{---}$	$.^{---}$	$.^{---}$	$.^{---}$	$.^{---}$	$.^{---}$	**$19^{38.9}$**	$.^{---}$
Pyrus communis agg.	4^{---}	9^{---}	$.^{---}$	$.^{---}$	$.^{---}$	$20^{14.4}$	$.^{---}$	**$38^{36.3}$**	6^{---}
Vicia hirsuta	1^{---}	4^{---}	$.^{---}$	$.^{---}$	$.^{---}$	$.^{---}$	$.^{---}$	**$24^{35.0}$**	7^{---}
Waldsteinio geoidis-Spiraeetum mediae									
Spiraea media	$.^{---}$	$.^{---}$	$.^{---}$	$.^{---}$	$.^{---}$	$.^{---}$	$.^{---}$	$.^{---}$	**$97^{98.4}$**
Waldsteinia geoides	$.^{---}$	$.^{---}$	$.^{---}$	$.^{---}$	$.^{---}$	$.^{---}$	$.^{---}$	$.^{---}$	**$36^{58.1}$**
Cotoneaster melanocarpus	$.^{---}$	1^{---}	$.^{---}$	$.^{---}$	$.^{---}$	$.^{---}$	$.^{---}$	$.^{---}$	**$31^{51.6}$**
Allium flavum	2^{---}	1^{---}	$.^{---}$	$.^{---}$	$.^{---}$	$.^{---}$	$.^{---}$	$.^{---}$	**$29^{48.1}$**
Cystopteris fragilis	$.^{---}$	$.^{---}$	$.^{---}$	$.^{---}$	$.^{---}$	$.^{---}$	$.^{---}$	$.^{---}$	**$25^{48.0}$**
Fallopia dumetorum	1^{---}	$.^{---}$	$.^{---}$	$.^{---}$	$.^{---}$	$.^{---}$	$.^{---}$	$.^{---}$	**$22^{43.8}$**
Iris variegata	$.^{---}$	$.^{---}$	$.^{---}$	$.^{---}$	$.^{---}$	$.^{---}$	$.^{---}$	$.^{---}$	**$21^{43.8}$**
Aconitum anthora	$.^{---}$	$.^{---}$	$.^{---}$	$.^{---}$	$.^{---}$	$.^{---}$	$.^{---}$	$.^{---}$	**$21^{43.8}$**
Cytisus hirsutus	$.^{---}$	$.^{---}$	$.^{---}$	$.^{---}$	$.^{---}$	$.^{---}$	$.^{---}$	$.^{---}$	**$19^{42.2}$**
Jovibarba globifera ssp. *hirta*	$.^{---}$	1^{---}	$.^{---}$	$.^{---}$	$.^{---}$	$.^{---}$	$.^{---}$	$.^{---}$	**$21^{41.8}$**
Trifolium alpestre	3^{---}	3^{---}	$.^{---}$	$.^{---}$	$.^{---}$	$.^{---}$	$.^{---}$	$.^{---}$	**$25^{41.4}$**
Seseli osseum	13^{---}	3^{---}	$.^{---}$	$.^{---}$	$.^{---}$	$.^{---}$	$.^{---}$	$.^{---}$	**$31^{40.1}$**
Phleum phleoides	7^{---}	3^{---}	$.^{---}$	$.^{---}$	$.^{---}$	$.^{---}$	$.^{---}$	$.^{---}$	**$26^{39.4}$**
Festuca stricta	1^{---}	12^{---}	$.^{---}$	$.^{---}$	$.^{---}$	$.^{---}$	$.^{---}$	$.^{---}$	**$28^{39.1}$**
Asplenium trichomanes	13^{---}	3^{---}	$.^{---}$	$.^{---}$	$.^{---}$	2^{---}	$.^{---}$	5^{---}	**$33^{39.0}$**
Linaria genistifolia agg.	1^{---}	$.^{---}$	$.^{---}$	$.^{---}$	$.^{---}$	$.^{---}$	$.^{---}$	$.^{---}$	**$17^{37.5}$**
Filipendula vulgaris	$.^{---}$	$.^{---}$	$.^{---}$	$.^{---}$	$.^{---}$	$.^{---}$	$.^{---}$	$.^{---}$	**$15^{37.4}$**
Euonymus verrucosus	$.^{---}$	$.^{---}$	$.^{---}$	$.^{---}$	$.^{---}$	6^{---}	$.^{---}$	$.^{---}$	**$21^{36.7}$**
Veronica spicata agg.	7^{---}	7^{---}	$.^{---}$	$.^{---}$	$.^{---}$	$.^{---}$	$.^{---}$	$.^{---}$	**$26^{36.3}$**
Poa pannonica	$.^{---}$	$.^{---}$	$.^{---}$	$.^{---}$	$.^{---}$	$.^{---}$	$.^{---}$	$.^{---}$	**$14^{35.6}$**
Elytrigia intermedia	$.^{---}$	1^{---}	$.^{---}$	$.^{---}$	$.^{---}$	$.^{---}$	$.^{---}$	$.^{---}$	**$15^{35.1}$**

Glechoma hirsuta	.$^{---}$	.$^{---}$	.$^{---}$	.$^{---}$	.$^{---}$	.$^{---}$	.$^{---}$	.$^{---}$	**12$^{33.8}$**
Campanula sibirica	.$^{---}$	.$^{---}$	.$^{---}$	.$^{---}$	.$^{---}$	.$^{---}$	.$^{---}$	.$^{---}$	**11$^{31.8}$**
Rostraria cristata	.$^{---}$	.$^{---}$	.$^{---}$	.$^{---}$	.$^{---}$	.$^{---}$	.$^{---}$	.$^{---}$	**11$^{31.8}$**
Festuca pseudodalmatica	.$^{---}$	.$^{---}$	.$^{---}$	.$^{---}$	.$^{---}$	.$^{---}$	.$^{---}$	.$^{---}$	**11$^{31.8}$**
Lactuca viminea	.$^{---}$	.$^{---}$	.$^{---}$	.$^{---}$	.$^{---}$	.$^{---}$	.$^{---}$	.$^{---}$	**11$^{31.8}$**
Galium intermedium agg.	1^{---}	1^{---}	.$^{---}$	.$^{---}$	.$^{---}$	.$^{---}$	.$^{---}$	.$^{---}$	**14$^{31.8}$**
**Species diagnostic for two associations**									
Hylotelephium maximum	**56$^{38.0}$**	12^{---}	.$^{---}$	4^{---}	.$^{---}$	.$^{---}$	.$^{---}$	5^{---}	**64$^{45.8}$**
Cotoneaster tomentosus	.$^{---}$	.$^{---}$	10^{---}	.$^{---}$	**67$^{46.4}$**	**61$^{41.3}$**	9^{---}	.$^{---}$	1^{---}
Viburnum lantana	.$^{---}$	24^{---}	15^{---}	.$^{---}$	**88$^{37.8}$**	**100$^{46.8}$**	.$^{---}$	76$^{29.7}$	8^{---}
Juniperus communis agg.	12^{---}	18^{---}	.$^{---}$	14^{---}	**58$^{31.1}$**	14^{---}	**73$^{43.3}$**	.$^{---}$	.$^{---}$
Lonicera alpigena	.$^{---}$	.$^{---}$	5^{---}	.$^{---}$	**62$^{51.4}$**	2^{---}	**45$^{33.5}$**	.$^{---}$	.$^{---}$
Crataegus monogyna agg.	14^{---}	22^{---}	5^{---}	.$^{---}$	8^{---}	**84$^{47.1}$**	.$^{---}$	**71$^{37.3}$**	15^{---}
Ligustrum vulgare	4^{---}	13^{---}	20^{---}	.$^{---}$	8^{---}	**71$^{36.5}$**	.$^{---}$	**95$^{55.4}$**	12^{---}
**Species of high fidelity, but with constancy ratio lower than 1.5**									
Cotoneaster integerrimus	91$^{29.8}$	90$^{29.2}$	10^{---}	21^{---}	29^{---}	29^{---}	55^{---}	81$^{23.3}$	14^{---}
Amelanchier ovalis	.$^{---}$	32^{---}	100$^{30.7}$	100$^{30.7}$	100$^{30.7}$	90$^{23.8}$	27^{---}	43^{---}	.$^{---}$
Sorbus aria agg.	22^{---}	31^{---}	85$^{25.0}$	11^{---}	100$^{35.2}$	80$^{21.4}$	82$^{22.9}$	5^{---}	17^{---}
Sesleria caerulea agg.	5^{---}	12^{---}	75$^{32.0}$	7^{---}	79$^{36.0}$	61$^{22.0}$	.$^{---}$	.$^{---}$	3^{---}
Berberis vulgaris	1^{---}	24^{---}	65$^{29.9}$	4^{---}	42$^{11.9}$	88$^{47.6}$	.$^{---}$	10^{---}	4^{---}
Corylus avellana	14^{---}	3^{---}	45$^{20.9}$	18^{---}	12^{---}	65$^{38.2}$	.$^{---}$	19^{---}	6^{---}
Rhamnus cathartica	14^{---}	24^{---}	20^{---}	.$^{---}$	46^{---}	86$^{42.8}$	.$^{---}$	48$^{14.1}$	22^{---}
Teucrium chamaedrys	2^{---}	47$^{7.3}$	20^{---}	.$^{---}$	8^{---}	71$^{32.4}$	.$^{---}$	43^{---}	64$^{26.7}$
Sorbus aucuparia	22^{---}	6^{---}	35$^{6.3}$	21^{---}	62$^{27.4}$	.$^{---}$	91$^{49.4}$	.$^{---}$	3^{---}
Rosa canina agg.	47$^{7.3}$	54$^{12.2}$	30^{---}	43^{---}	.$^{---}$	6^{---}	18^{---}	100$^{44.4}$	33^{---}
Ribes alpinum	2^{---}	1^{---}	.$^{---}$	.$^{---}$	8^{---}	6^{---}	36$^{27.0}$	48$^{39.3}$	.$^{---}$
Galium glaucum	15^{---}	38$^{28.6}$	.$^{---}$	.$^{---}$	.$^{---}$	.$^{---}$	.$^{---}$	5^{---}	46$^{36.9}$
Frangula alnus	1^{---}	9^{---}	35$^{30.9}$	36$^{31.7}$	.$^{---}$	.$^{---}$	.$^{---}$	.$^{---}$	1^{---}
Rubus saxatilis	.$^{---}$	.$^{---}$	30$^{34.5}$	.$^{---}$	25$^{27.1}$	.$^{---}$	.$^{---}$	.$^{---}$	.$^{---}$
Melittis melissophyllum agg.	.$^{---}$	1^{---}	.$^{---}$	.$^{---}$	29$^{34.5}$	20$^{21.3}$	.$^{---}$	.$^{---}$	1^{---}
Rhamnus alpina	.$^{---}$	.$^{---}$	.$^{---}$	.$^{---}$	71$^{47.5}$	53$^{31.4}$	36$^{16.5}$	.$^{---}$	.$^{---}$
**The most common accompanying species**									
Quercus petraea agg.	22^{---}	9^{---}	.$^{---}$	57$^{22.1}$	38^{---}	63$^{26.8}$	.$^{---}$	52$^{18.5}$	10^{---}
Hypnum cupressiforme agg. d	32$^{14.8}$	9^{---}	20^{---}	39$^{22.0}$	4^{---}	.$^{---}$	.$^{---}$	33$^{16.4}$	4^{---}
Polygonatum odoratum	43^{---}	24^{---}	40^{---}	18^{---}	42^{---}	49$^{11.0}$	.$^{---}$	52^{---}	35^{---}
Dicranum scoparium d	5^{---}	6^{---}	15^{---}	18$^{19.0}$	.$^{---}$	.$^{---}$	.$^{---}$	.$^{---}$	4^{---}
Carex humilis	3^{---}	29$^{20.6}$	15^{---}	4^{---}	4^{---}	10^{---}	.$^{---}$	14^{---}	17^{---}
Euphorbia cyparissias	47$^{15.2}$	60$^{25.0}$	25^{---}	11^{---}	.$^{---}$	12^{---}	.$^{---}$	38^{---}	53$^{19.3}$
Hypericum perforatum	29$^{17.1}$	29$^{17.1}$	.$^{---}$	4^{---}	.$^{---}$	.$^{---}$	.$^{---}$	14^{---}	38$^{25.4}$
Anthericum ramosum	7^{---}	29$^{14.8}$	25^{---}	.$^{---}$	29^{---}	10^{---}	.$^{---}$	.$^{---}$	26$^{11.9}$
Brachypodium pinnatum agg.	7^{---}	34$^{22.0}$	20^{---}	.$^{---}$	4^{---}	10^{---}	.$^{---}$	14^{---}	22^{---}
Origanum vulgare	11^{---}	18^{---}	.$^{---}$	.$^{---}$	8^{---}	14^{---}	.$^{---}$	5^{---}	22$^{16.5}$
Pinus sylvestris	12^{---}	13^{---}	5^{---}	11^{---}	42$^{30.6}$	29$^{16.8}$	.$^{---}$	.$^{---}$	.$^{---}$
Galium mollugo agg.	4^{---}	6^{---}	5^{---}	11^{---}	42$^{23.3}$	22^{---}	.$^{---}$	24^{---}	33$^{15.6}$
Rosa spinosissima	.$^{---}$	7^{---}	.$^{---}$	.$^{---}$	29$^{14.8}$	20^{---}	.$^{---}$	33$^{18.9}$	36$^{21.6}$
Lonicera xylosteum	4^{---}	6^{---}	25^{---}	.$^{---}$	46$^{20.1}$	55$^{27.8}$	18^{---}	38$^{13.7}$	1^{---}

Campanula rotundifolia agg.	20^{---}	21^{---}	15^{---}	25^{---}	8^{---}	4^{---}	$.^{---}$	5^{---}	12^{---}
Solidago virgaurea agg.	$19^{14.8}$	3^{---}	$30^{29.4}$	11^{---}	$.^{---}$	2^{---}	$.^{---}$	$.^{---}$	3^{---}
Populus tremula	3^{---}	$.^{---}$	$20^{24.6}$	$11^{9.5}$	$.^{---}$	$.^{---}$	9^{---}	$.^{---}$	$.^{---}$
Seseli libanotis	2^{---}	6^{---}	$.^{---}$	$.^{---}$	$17^{19.6}$	$14^{15.7}$	$.^{---}$	$.^{---}$	3^{---}
Hypericum montanum	3^{---}	1^{---}	$.^{---}$	$.^{---}$	8^{---}	$8^{12.4}$	$.^{---}$	$.^{---}$	1^{---}
Acer campestre	3^{---}	6^{---}	$.^{---}$	$.^{---}$	$.^{---}$	$20^{24.5}$	$.^{---}$	10^{---}	6^{---}
Fagus sylvatica	2^{---}	12^{---}	$30^{24.1}$	$.^{---}$	$29^{23.1}$	10^{---}	$.^{---}$	$.^{---}$	1^{---}
Convallaria majalis	3^{---}	1^{---}	$15^{17.9}$	$.^{---}$	$17^{20.8}$	$.^{---}$	$.^{---}$	$.^{---}$	3^{---}
Mercurialis perennis	5^{---}	6^{---}	$.^{---}$	$.^{---}$	$21^{26.0}$	6^{---}	$.^{---}$	5^{---}	$.^{---}$
Acer pseudoplatanus	4^{---}	3^{---}	$40^{26.8}$	$.^{---}$	$33^{20.1}$	$.^{---}$	$36^{23.2}$	$.^{---}$	3^{---}
Poa nemoralis	$39^{27.4}$	10^{---}	$.^{---}$	11^{---}	$.^{---}$	$.^{---}$	$.^{---}$	29^{---}	24^{---}
Silene nutans	$20^{20.4}$	9^{---}	$.^{---}$	11^{---}	$.^{---}$	$.^{---}$	$.^{---}$	10^{---}	4^{---}
Polypodium vulgare	$19^{14.3}$	4^{---}	$.^{---}$	4^{---}	$.^{---}$	2^{---}	$.^{---}$	14^{---}	$26^{23.9}$
Fragaria vesca	9^{---}	4^{---}	$30^{26.5}$	$.^{---}$	4^{---}	12^{---}	$.^{---}$	5^{---}	11^{---}
Genista tinctoria	$14^{18.1}$	1^{---}	10^{---}	$.^{---}$	4^{---}	$.^{---}$	$.^{---}$	$.^{---}$	4^{---}
Tilia platyphyllos	2^{---}	3^{---}	10^{---}	$.^{---}$	4^{---}	8^{---}	$.^{---}$	$.^{---}$	4^{---}
Bupleurum falcatum	3^{---}	$22^{11.9}$	$.^{---}$	$.^{---}$	$.^{---}$	$37^{28.0}$	$.^{---}$	24^{---}	14^{---}
Silene viscaria	$21^{21.0}$	4^{---}	$.^{---}$	4^{---}	$.^{---}$	$.^{---}$	$.^{---}$	5^{---}	$22^{22.7}$
Clematis vitalba	$.^{---}$	4^{---}	5^{---}	$.^{---}$	4^{---}	8^{---}	$.^{---}$	$14^{17.4}$	1^{---}
Hedera helix	5^{---}	3^{---}	$.^{---}$	4^{---}	$.^{---}$	$22^{27.5}$	$.^{---}$	10^{---}	1^{---}
Dactylis glomerata agg.	2^{---}	1^{---}	5^{---}	$.^{---}$	$.^{---}$	$.^{---}$	$.^{---}$	10^{---}	$17^{22.9}$
Tanacetum corymbosum	7^{---}	10^{---}	$.^{---}$	$.^{---}$	$.^{---}$	2^{---}	$.^{---}$	10^{---}	$21^{22.9}$
Geranium sanguineum	21^{---}	25^{---}	$.^{---}$	$.^{---}$	$.^{---}$	14^{---}	$.^{---}$	19^{---}	$38^{24.9}$
Fragaria viridis	15^{---}	$22^{15.7}$	$.^{---}$	$.^{---}$	$.^{---}$	$.^{---}$	$.^{---}$	24^{---}	$19^{12.6}$
Cytisus scoparius	$13^{6.3}$	3^{---}	$.^{---}$	$25^{22.2}$	$.^{---}$	$.^{---}$	$.^{---}$	$29^{26.9}$	$.^{---}$
