# Supplementary material for: Differentiation of natural scrub communities of the Cotoneastro-Amelanchieretum group in Central Europe
Source: PLoS One. 2022 Apr 12;17(4):e0266868. doi: 10.1371/journal.pone.0266868 (PMC9004749; doi:10.1371/journal.pone.0266868)
Supplement: S6 Appendix — (PDF) [file pone.0266868.s006.pdf]

Differentiation of natural scrub communities of *Cotoneastro-Amelanchieretum* group in Central Europe

Świerkosz K., Reczyńska K.

Appendix S6

Detailed description of shrub communities from the *Cotoneastro-Amelanchieretum* group and relatives

Shrub communities from *Cotoneastro-Amelanchieretum* s. lato

Cluster 1. ***Cytiso scoparii-Cotoneasteretum integerrimi* Stöcker 1962** (incl. *Cotoneastero integerrimi-Polygonatetum odorati* Firbas et Sigmond 1928 (§ 2b, nomen nudum), *Lembotropido-Cotoneasteretum* Rauschert 1990, *Polygonato-Sorbetum ariae* Kolbek et Petříček 1985 prov (§ 3b)).

Orig: Stöcker (1962, p. 926): *Scopario-Cotoneasteretum* ass. nova.

Name-giving taxa: *Sarothamnus scoparius* [=*Cytisus scoparius*], *Cotoneaster integerrimus*

Holotypus: hoc loco, Stöcker 1962, Tabelle 10, Auf. 9.

Number of relevés: 95

Diagnostic species: *Asplenium septentrionale*, *Aurinia saxatilis*, *Calamagrostis arundinacea*, *Festuca pallens*, *Hieracium schmidtii*, *Hylotelephium maximum*, *Jovibarba globifera* ssp. *globifera*, *Lembotropis nigricans*, *Vincetoxicum hirundinaria*

Constant species: *Cotoneaster integerrimus*, *Euphorbia cyparissias*, *Festuca pallens*, *Hylotelephium maximum*, *Polygonatum odoratum*, *Rosa canina* agg., *Vincetoxicum hirundinaria*

Distribution: **Bohemian Massif**: Czech Republic, Poland, Germany.

Ecology: bedrocks of almost lime-free, yet moderately nutrient-rich (basalt, greenschist, trachyte, andesite, diabase, some kinds of shales); between 100 m and 670 m.a.s.l.

Nomenclatural note:

Common occurrence of *Cytisus scoparius* in *locus classicus* is a local phenomenon rather than a general rule. In the analyzed set of data this species has no diagnostic value (Table 1, col. 1). However, most of the species occurred in the Table 10 in Stöcker [1] are common in other sites of this association, and also the ecological character of this species assemblage looks to be distinct and coherent. All the Stöcker's [1] relevés belong to this cluster.

Cluster 2. ***Cotoneastro integerrimi-Amelanchieretum ovalis* Faber ex Th. Müller 1966** (incl. *Cotoneaster-Amelanchier*-Gesträuch Faber 1936 (§ 3c), *Junipero communis-Cotoneasteretum integerrimi* Hofmann 1958, *Seslerio-Cotoneastretum* Rauschert (1969) 1990, *Roso ellipticae-Cotoneasteretum* Rauschert 1990, *Erysimo-Amelanchieretum* Rauschert 1990)

Orig. Müller (1966): *Cotoneastro-Amelanchieretum* Faber ex Th. Müller 1966, after Rennwald et al. (2000) as *nom*. *cons*.

Number of relevés: 68

Diagnostic species: *Anemone sylvestris*, *Asperula cynanchica*, *Centaurea scabiosa*, *Cirsium acaulon*, *Erysimum crepidifolium*, *Salvia pratensis*, *Scabiosa columbaria*, *Thymus pulegioides* agg.

Constant species: *Cotoneaster integerrimus*, *Euphorbia cyparissias*, *Rosa canina* agg., *Teucrium chamaedrys*

Distribution: **Central Europe**: Germany, Czech Republic, Austria, Poland, Hungary, Slovakia.

Ecology: calcicolous community, on rocks rich in lime (dolomite, limestone, marble); between 100 m and 575 m (max. 700 m) a.s.l.

Cluster 3. ***Erico carneae-Amelanchieretum* ovalis Passarge 1997** (incl. *Erica herbacea-Frangula alnus* Gesellschaft Passarge 1997)

Orig: Passarge (1997, p. 46-47), *Erico-Amelanchieretum ovalis* ass. nova.

Holotypus: Passarge 1997, Tabelle 6 Aufhname Nr. 9.

Number of relevés: 20

Diagnostic species: *Bromus erectus*, *Buphthalmum salicifolium*, *Calamagrostis varia*, *Carex alba*, *C. flacca*, *C. ornithopoda*, *Cephalanthera damasonium*, *Erica carnea*, *Festuca ovina* agg., *Galium anisophyllon*, *Globularia cordifolia*, *Hepatica nobilis*, *Hippocrepis comosa*, *Laserpitium siler*, *Lotus corniculatus* agg., *Melica nutans*, *Molinia caerulea* agg., *Phyteuma orbiculare*, *Pinus mugo*, *Polygala chamaebuxus*, *Potentilla erecta*, *Prenanthes purpurea*, *Ranunculus polyanthemos*, *Rhamnus saxatilis*, *Rubus saxatilis*, *Salvia glutinosa*, *Pseudoscleropodium purum*, *Teucrium montanum*, *Tortella tortuosa*, *Trifolium montanum*, *Valeriana tripteris*

Constant species: *Amelanchier ovalis*, *Berberis vulgaris*, *Corylus avellana*, *Erica carnea*, *Galium anisophyllon*, *Melica nutans*, *Picea abies*, *Pinus mugo*, *Polygala chamaebuxus*, *Potentilla erecta*, *Ranunculus polyanthemos*, *Sesleria caerulea* agg., *Sorbus aria* agg.

Distribution: **Eastern Alps** (Austria, Germany)

Ecology: mountain, calcicolous community occurring on consolidate, shallow rendzinas at altitudes between (520 m) 950 m and 1100 m.a.s.l.

Cluster 4. ***Calluno vulgaris-Amelanchieretum ovalis* Rauschert (1969) 1990** (incl. *Cotoneastero-Amelanchieretum* Korneck 1974 p.p.)

Orig: Rauschert (1990, p. 210-212 & Tab. 1): *Calluno-Amelanchieretum* Rauschert (69) ass. nov.

Holotypus: Rauschert (1990), Tab. 1 releve Nr. 3.

Number of relevés: 28

Diagnostic species: *Agrostis vinealis*, *Avenella flexuosa*, *Betula pendula*, *Calluna vulgaris*, *Ceratodon purpureus*, *Festuca heteropachys*, *F. lemanii*, *Frangula alnus*, *Genista pilosa*, *Hieracium umbellatum*, *Polytrichum piliferum*, *Rubus fruticosus* agg., *Rumex acetosella*, *Teucrium scorodonia*

Constant species: *Amelanchier ovalis*, *Avenella flexuosa*, *Calluna vulgaris*, *Genista pilosa*, *Polytrichum piliferum*, *Quercus petraea* agg., *Rosa canina* agg., *Teucrium scorodonia*

Distribution: **central Germany**.

Ecology: acidophilous and poor in nutrients bedrocks (Devonian and Ordovician clay shales, porphyry), between 190 m and 450 m.a.s.l.

Cluster 5. **comm. *Cotoneaster tomentosus-Amelanchier ovalis sensu* Moor 1979** (incl. *Cotoneastro-Amelanchieretum* var with *Rhamnus saxatilis* and *Cotoneastro-Amelanchieretum* var. with *Colutea arborescens* Moor 1979; *Cotoneastro tomentosi-Amelanchieretum* Moor 1979 sensu Oberdorfer & Müller 1992, *Coluteo arborescentis-Amelanchieretum ovalis* (Moor 1979) de Foucault & Julve, nomen nudum; *Rhamno alpinae-Amelanchieretum ovalis* de Foucault & Julve, ass. nova. 2001, nomen nudum; non *Cotoneastro tomentosi-Amelanchieretum* Jakucs 1961)

Number of relevés: 24

Diagnostic species: *Arctostaphylos uva-ursi*, *Carduus defloratus* agg., *Cotoneaster tomentosus*, *Gentiana lutea*, *Juniperus communis* agg., *Laburnum anagyroides*, *Laserpitium latifolium*, *Leucanthemum vulgare* agg., *Lonicera alpigena*, *Melittis melissophyllum* agg., *Sorbus*. *mougeotii*, *Taxus baccata*, *Viburnum lantana*

Constant species: *Amelanchier ovalis*, *Berberis vulgaris*, *Carduus defloratus* agg., *Cotoneaster tomentosus*, *Galium mollugo* agg., *Juniperus communis* agg., *Leucanthemum vulgare* agg., *Lonicera alpigena*, *L. xylosteum*, *Pinus sylvestris*, *Polygonatum odoratum*, *Rhamnus alpina*, *Rhamnus cathartica*, *Rosa pendulina*, *Sesleria caerulea* agg., *Sorbus aria* agg., *S. aucuparia*, *S. mougeotii*, *Viburnum lantana*

Distribution**: Jura Mts (**France and Switzerland)

Ecology: calcicolous shrubs of south-facing slopes, between 450 m and 860 m.a.s.l.

Nomenclatural note. Original description by Moor [2] placed this community in the *Cotoneastro-Amelanchiretum* s. lato, as two distinct subassociations. From this reason, and due to the scarce

phytosociological material, we did not decide to describe new association and leave this issue for further analysis based on wider material.

Relevés originally assigned to the ass. *Cotoneastro-Amelanchieretum*, but not belonging to this association

Cluster 6. ***Coronillo emeri-Prunetum mahaleb*** **Gallandat 1972**

Number of relevés: 49

Diagnostic species: *Berberis vulgaris*, *Cornus sanguinea*, *Corylus avellana*, *Cotoneaster tomentosus*, *Crataegus laevigata* agg., *C. monogyna* agg., *Fraxinus excelsior*, *Hippocrepis emerus*, *Ligustrum vulgare*, *Malus sylvestris* agg., *Prunus mahaleb*, *Rhamnus cathartica*, *Rosa glauca*, *Teucrium chamaedrys*, *Viburnum lantana*, *Viola hirta*

Constant species: *Amelanchier ovalis*, *Berberis vulgaris*, *Cornus sanguinea*, *Corylus avellana*, *Cotoneaster tomentosus*, *Crataegus laevigata* agg., *C. monogyna* agg., *Fraxinus excelsior*, *Hippocrepis emerus*, *Ligustrum vulgare*, *Lonicera xylosteum*, *Malus sylvestris* agg., *Polygonatum odoratum*, *Prunus mahaleb*, *P. spinosa*, *Quercus petraea* agg., *Rhamnus alpina*, *R. cathartica*, *Rosa glauca*, *Sorbus aria* agg., *Teucrium chamaedrys*, *Viburnum lantana*, *Vincetoxicum hirundinaria*, *Viola hirta*

Distribution: relevés belonging to this association, originally classified as *Cotoneastro-Amelanchieretum* come from Switzerland and France (Moor 1979, 45 relevés) and Austria (4 relevés from NSG Glaslauterriegel-Heferlberg).

Ecology: calcicolous and xerothermic community occurring at altitudes between 400 m and 820 m.a.s.l. on south-facing slopes (S, SW).

Associations with the regular occurrence of *Cotoneaster integerrimus* and/or *Amelanchier ovalis*, outside the range of *Cotoneastro-Amelanchieretum* s. lato

Cluster 7. ***Cotoneastro integerrimi-Sorbetum chamaemespili*** Gillet in Gallandat et al. 1995

Number of relevés: 11

Diagnostic species: *Daphne mezereum*, *Juniperus communis* agg., *Lonicera alpigena*, *L. caerulea*, *L. nigra*, *Picea abies*, *Rosa pendulina*, *Rubus idaeus*, *Salix appendiculata*, *Salix caprea*, *Sambucus racemosa*, *Sorbus chamaemespilus*

Constant species: *Cotoneaster integerrimus*, *Daphne mezereum*, *Juniperus communis* agg., *Lonicera alpigena*, *L. caerulea*, *L. nigra*, *Picea abies*, *Rosa pendulina*, *Salix appendiculata*, *Sorbus aria* agg., *S. aucuparia*, *S. chamaemespilus*

Distribution: **Alps** in Switzerland and on the German/Austrian border

Ecology: on calcicolous lapiaz, sometimes also in spruce stands on lapiaz and in rocky pastures. It is therefore a primarily heliophilous and relatively thermophilic association, mainly on the south-facing slopes between 900 m and 1350 m.a.s.l.

Cluster 8. ***Pruno spinosae-Ligustretum vulgaris* Tüxen 1952 subass. with *Cotoneaster integerrimus* Korneck 1974**

Number of relevés: 21

Diagnostic species: *Crataegus monogyna* agg., *Euonymus europaeus*, *Ligustrum vulgare*, *Prunus avium*, *P. spinosa*, *Pyrus communis* agg., *Ribes alpinum*, *Rosa canina* agg., *Vicia hirsuta*

Constant species: *Amelanchier ovalis*, *Cotoneaster integerrimus*, *Crataegus monogyna* agg., *Euonymus europaeus*, *Ligustrum vulgare*, *Polygonatum odoratum*, *Prunus spinosa*, *Quercus petraea* agg., *Rhamnus cathartica*, *Ribes alpinum*, *Rosa canina* agg., *Teucrium chamaedrys*, *Viburnum lantana*

Distribution: analyzed relevés with the high share of *C. integerrimus* and *A. ovalis* come from **Germany** and **France**. However, the range of the association – including its full variability – covers the whole of Central Europe.

Ecology: association common in lowlands and colline landscapes in Central Europe, over a broad range of mesic and dry habitats, mainly developed as secondary scrub. However, in some rocky sites, it is presumably a natural type of vegetation.

Cluster 9. ***Waldsteinio geoidis-Spiraeetum mediae* Zólyomi 1936**

Number of relevés: 71

Diagnostic species: *Aconitum anthora*, *Allium flavum*, *Asplenium trichomanes*, *Campanula sibirica*, *Cotoneaster melanocarpus*, *Cystopteris fragilis*, *Cytisus hirsutus*, *Elytrigia intermedia*, *Euonymus verrucosus*, *Fallopia dumetorum*, *Festuca pseudodalmatica*, *F. stricta*, *Filipendula vulgaris*, *Galium intermedium* agg., *Glechoma hirsuta*, *Hylotelephium maximum*, *Iris variegata*, *Jovibarba globifera* ssp.

hirta, *Lactuca viminea*, *Linaria genistifolia* agg., *Phleum phleoides*, *Poa pannonica*, *Rostraria cristata*, *Seseli osseum*, *Spiraea media*, *Trifolium alpestre*, *Veronica spicata* agg., *Waldsteinia geoides*
Constant species: *Euphorbia cyparissias*, *Galium glaucum*, *Hylotelephium maximum*, *Spiraea media*, *Teucrium chamaedrys*, *Vincetoxicum hirundinaria*

Distribution: **Western part of Central Europe:** Hungary, Slovakia, Austria

Ecology: community of dry and rocky habitats, on rendzinas or rankers (andesite) soils. Usually occurs at altitude from 300 m to 750 m.a.s.l.. In Hungary described also from forest edges or even ruderal habitats, with high participation of nitrophilous species.

References

1. Stöcker G. Vorarbeit zu einer Vegetationsmonographie des Naturschutzgebietes Bodetal. I. Offene Pflanzengesellschaften. Wiss. Z. Martin-Luther-Univ. Halle-Wittenberg. Math.-Naturwiss. Reihe. 1962;11: 897–936.

2. Moor M. *Cotoneastro-Amelanchieretum*, a natural cover community in the Jura. Phytocoenologia. 1979;6: 388–402.
